# The association between Parkinson’s disease and autoimmune diseases: A systematic review and meta-analysis

**DOI:** 10.3389/fimmu.2023.1103053

**Published:** 2023-01-25

**Authors:** Mingqiang Li, Juan Wan, Zhenhong Xu, Beisha Tang

**Affiliations:** ^1^ Department of Neurology, The First Affiliated Hospital, Multi-Omics Research Center for Brain Disorders, Hengyang Medical School, University of South China, Hengyang, Hunan, China; ^2^ The First Affiliated Hospital, Clinical Research Center for Immune-Related Encephalopathy of Hunan Province, Hengyang Medical School, University of South China, Hengyang, Hunan, China; ^3^ Department of Neurology, Xiangya Hospital, Central South University, Changsha, Hunan, China

**Keywords:** Parkinson’s disease, autoimmune disease, bullous pemphigoid, inflammatory bowel disease, comorbidity, Crohn ‘s disease, ulcerative colitis

## Abstract

Parkinson’s disease (PD) is a neurodegenerative disorder that frequently occurs in the older population. Previous epidemiological studies have suggested an association between PD and autoimmune diseases (AIDs). However, some studies have shown conflicting results. This study aimed to summarize existing epidemiological studies on the association between PD with AIDs and to conduct a meta-analysis of combinable results. Four electronic databases (PubMed, Embase, Web of Science Core Collection, and MEDLINE) were searched from each database’s inception date until December 12, 2022. All studies that explored the relationship between PD and AIDs were included for quantitative analysis and qualitative review. The pooled relative risk with 95% confidence intervals (CIs) was calculated using a random or fixed effects model. A total of 46 observational studies involving 873,643 patients and 13,402,821 controls were included; ultimately, 38 studies were included in the meta-analysis. The risk of PD combined with AIDs was significantly higher (odds ratio [OR]=1.55, 95% CI: 1.33–1.81), and subgroup analysis found no significant differences in risk by study type, gender, age, and race. Regarding the AID types, the results showed an increased risk of PD combined with bullous pemphigoid (OR=2.67, 95% CI: 2.15–3.31), inflammatory bowel disease (OR=1.30, 95% CI: 1.18–1.45), Crohn’s disease (OR=1.30, 95% CI: 1.20–1.42), ulcerative colitis (OR=1.31, 95% CI: 1.14–1.50), Sjögren’s syndrome (OR=1.61, 95% CI: 1.24–2.09), and Graves’ disease (OR=1.45, 95% CI: 1.24–1.70) than controls. However, there appeared to be no significant association between PD and systemic lupus erythematosus (OR=0.82, 95% CI: 0.66–1.03), multiple sclerosis (OR=2.02, 95% CI: 0.87–4.70), rheumatoid arthritis (OR=0.79, 95% CI: 0.61–1.03), or celiac disease (OR=1.16, 95% CI: 0.79–1.69). This study supports the existence of a strong link between AIDs and PD. When PD and AIDs are identified, clinicians need to be aware of the possibility of coexistence. However, there are some limitations of this study, such as the apparent heterogeneity of some of the results and the fact that most of the included study types were retrospective. Therefore, future larger prospective cohort studies are needed to further explore the interaction between PD and AIDs.

**Systematic review registration:**

INPLASY, identifier INPLASY202280088.

## 1 Introduction

Parkinson’s disease (PD) is a common neurodegenerative movement disorder with symptoms including resting tremor, bradykinesia, rigidity, and postural disturbances, as well as many non-motor symptoms (1, 2). PD affects 1–2/1,000 people, and its prevalence increases with age (3–5). The etiology and pathogenesis of PD are complex and are currently thought to be due to a combination of genetic, environmental, and aging factors (4, 6). There is growing evidence implicating immune dysfunction in the etiology of PD, and it has even been proposed that PD may be an autoimmune disease (AID) (7–9).

Genetic and epidemiological studies have linked AIDs to PD. AIDs and PD were foaund to share a common genetic pathway, suggesting that the immune system influences the pathogenesis of PD (10, 11). Moreover, several epidemiological studies have observed an association between PD and AIDs (12–16). However, two of these large population-based studies reached different conclusions, with Rugbjerg et al. (13) reporting a risk association between 32 AIDs and PD, but not associations between AIDs and subsequent PD risk. Li et al. (12) reported a risk association between 33 AIDs and PD, which suggested an increased risk of combined PD in patients with AIDs. However, conclusions drawn for a single AID type are also inconsistent; for example, Chang et al. (14) Chen et al. (16), and Wu et al. (17) suggest an increased risk of PD in combination with Sjögren’s syndrome (SS), while Rugbjerg et al. (13) and Li et al. (12) suggest no significance.

In summary, there are shared mechanisms between PD and many AIDs, with PD often occurring in conjunction with at least one AID. However, epidemiological studies involving PD and AIDs have yielded inconsistent results, with contradictory and controversial findings. Therefore, To address these issues, we carefully searched four commonly used databases and performed a systematic review and meta-analysis of published clinical studies investigating the relationship between PD and some common AIDs. This will help us to clarify the relationship between PD and AIDs and to provide a reference for research on the pathogenesis of PD.

## 2 Materials and methods

### 2.1 Search strategy

This study was performed in accordance with the guidelines of the Preferred Reporting Items for Systematic Reviews and Meta-Analysis (PRISMA) program. This protocol was registered in INPLASY (registration number: INPLASY202280088).

The PubMed, Embase, Web of Science Core Collection, and MEDLINE databases were searched for literature describing a link between PD and AIDs. From the day that each database was created until December 12, 2022, each database was searched. PD and AID-related free-text phrases were combined with restricted vocabulary terms that were particular to the database. To capture other potentially relevant articles, we also combined search terms representing PD with search terms for 34 AIDs (i.e., chronic inflammatory demyelinating polyneuropathy, Guillain–Barre syndrome, multiple sclerosis [MS], myasthenia gravis [MG], Addison’s disease, type 1 diabetes mellitus [T1D], Graves’ disease [GD], Hashimoto disease, autoimmune hepatitis, inflammatory bowel disease [IBD], Crohn’s disease [CD], ulcerative colitis [UC], coeliac disease [CLD], pernicious anemia [PA], primary biliary cirrhosis [PBC], alopecia areata, primary sclerosing cholangitis, antiphospholipid syndrome, autoimmune hemolytic anemia [AIHA], immune thrombocytopenic purpura, polymyositis [PM], rheumatoid arthritis [RA], polyarteritis nodosa, giant cell arteritis, bullous pemphigoid [BP], discoid lupus erythematosus, vitiligo, Behcet’s disease [BD], scleroderma, SS, systemic lupus erythematosus [SLE], dermatitis herpetiformis, narcolepsy, and rheumatic fever) that were reported to be most prevalent (i.e., with a worldwide prevalence rate of ≥10/100,000 people based on a review that included a comprehensive list of AIDs) (18). To be as comprehensive as possible, the search was not restricted to any study type. An example search strategy for the PubMed database is described in **Supplementary Table 1**.

### 2.2 Inclusion criteria and study selection

Peer-reviewed publications that presented population-based research showing a link between PD and any form of AIDs were required for the study’s inclusion. Case reports and case series were not included since it was unclear how well they sampled from among those groups. To prevent duplication and incorrect weighting toward more frequently referenced or discussed articles, review papers, meta-analyses, organizational recommendations, editorial letters, and professional views were eliminated. Additionally, conference abstracts were disregarded since their full study reports could not be obtained for evaluation and their scientific rigor had not undergone peer assessment. Additionally, only research that appeared in English-language publications was considered.

The four databases’ search results yielded articles that were all imported into Endnote for review. For study inclusion, there were two rounds of screening. Two reviewers (ML and JW) independently performed screening in the first round by examining titles, abstracts, and key terms for relevance to both AIDs and PD. The entire contents of the articles found during the first round of screening were collected and carefully read to determine eligibility in the second round of screening. To establish agreement, potential disagreements throughout the study selection process were discussed with a third reviewer (ZX).

### 2.3 Data extraction

Data from included studies were extracted into a standard table, detailing authors, year of publication, country, study period, study design, selection of control and comparison groups, number of study participants, reported risk estimates for PD associated with AIDs, and any adjustments for confounding factors in producing effect estimates.

### 2.4 Quality assessment

Study quality was evaluated on the Newcastle–Ottawa Scale (NOS). Studies that achieve seven or more stars on the NOS are considered high quality, while four to six stars indicate moderate quality, and less than four stars indicate poor quality.

### 2.5 Meta-analysis

Meta-analysis was performed for each study reporting the correlation between PD and AID. Statistical heterogeneity between studies was assessed for each outcome by examining study-specific effect size and heterogeneity (I^2^) statistics. I^2^ values of <25%, 25–50%, 51–75%, and >75% were considered to denote no, mild, moderate, and large heterogeneity, respectively (19). In meta-analyses of multiple studies for a specific outcome, a fixed-effect estimate was calculated if the I^2^ value was <50%; a random-effect estimate was calculated if the I^2^ value was ≥50%. Forest plots were constructed to present risk estimates and meta-analysis results for each AID reported by at least three studies to be associated with PD. In meta-analyses of cohort studies, the hazard ratio (HR) and standardized incidence ratio (SIR) were treated as being equal to the odds ratio (OR). Publication bias was assessed by observing the symmetry of the funnel plot and performing Egger’s and Begg’s tests.

Statistical analysis was performed using STATA software (Version 16.0, StataCorp, College Station, TX, USA). Statistical significance was set at P<0.05.

## 3 Results

### 3.1 Study selection

**Figure 1:**
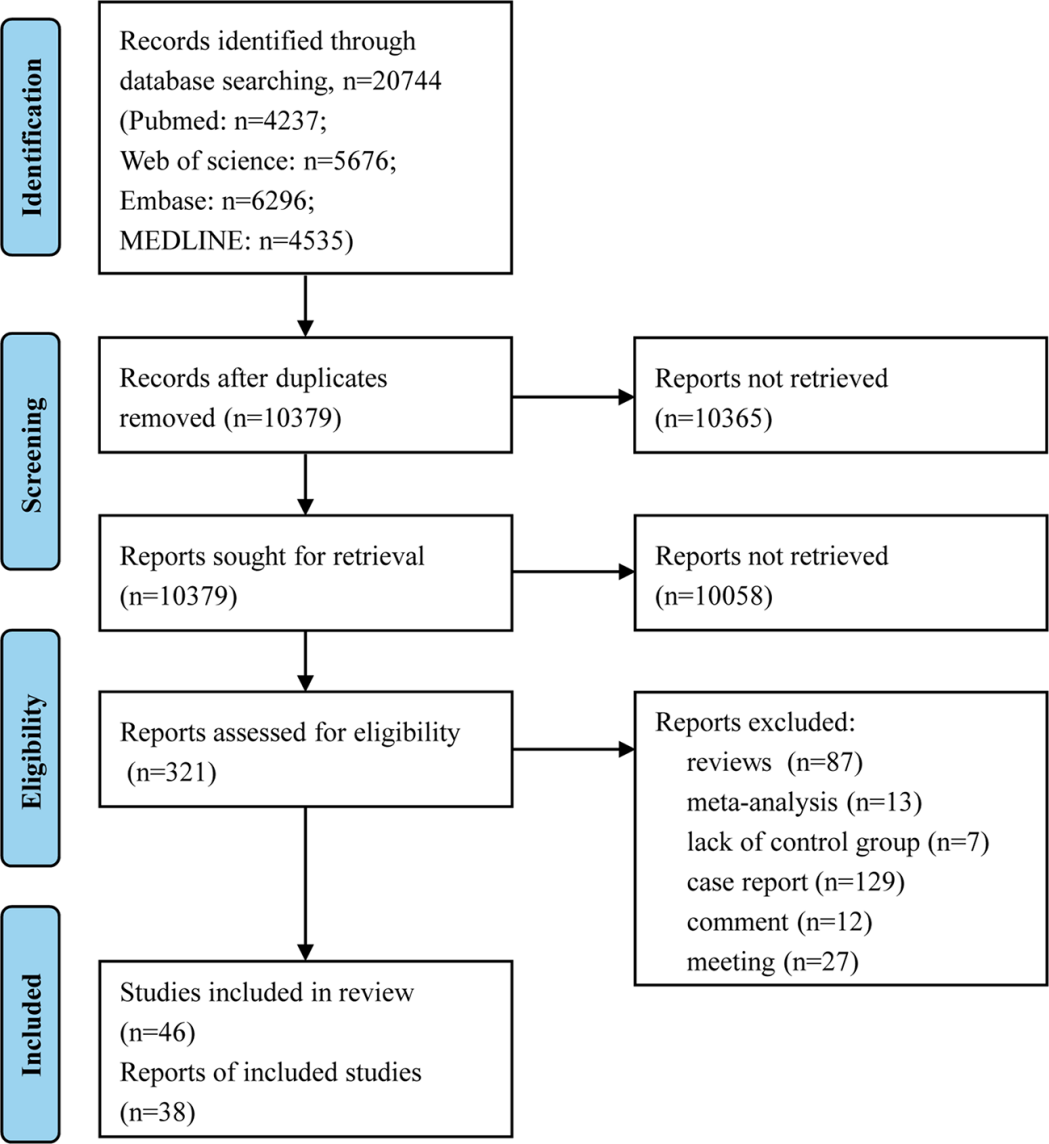
Flow chart for study inclusion and exclusion process.

In the original search, 4,535 results from MEDLINE, 4,237 from PubMed, 5,676 from the Web of Science Core Collection, and 6,296 from Embase were found. The four databases’ search results yielded articles that were all imported into Endnote for screening. There were 10,379 papers uploaded to Endnote for first-stage screening following the removal of duplicates. Following the initial screening of publications by looking at their titles, abstracts, and key words relating to PD or AIDs, 10,058 publications were disqualified for not addressing both PD and AIDs, and 321 papers were found to be potentially relevant and subsequently evaluated for eligibility. After the first round, 275 articles were excluded for being a review article or meta-analysis (n=100), not having a control group (n=7), being a comment (n=12), a case report (n=129), or a meeting (n=27). Finally, 46 articles met all the inclusion criteria and were included in this review, 38 of which were included for quantitative synthesis for having calculable risk estimates (**Figure 1**).

### 3.2 Study characteristics

**Table 1** shows a detailed description of the key characteristics of the 46 included studies. In brief, there were 16 national studies; 18 (39.1%) were conducted in European populations, 5 (10.9%) were conducted in South America, and 23 (50.0%) were conducted in Asia. In terms of study design, 4 (8.5%) were cross-sectional studies, 18 (38.3%) were case-control studies, and 25 (53.2%) were cohort (including 8 prospective cohort and 17 retrospective cohort) studies, with the study by Brick et al. (29) reporting both case-control and retrospective cohort studies.

**Table 1:** Characteristics of included studies.

Study	Country	Study period	Study design	Cases (events/total)	Controls (events/total)	Study population	Matched control group
Cases group for Parkinson’s diseases							
Rugbjerg et al. (13)	Denmark	1986-2006	Case-control study	Total (13695); IBD (52), CD (10), UC (42); SS (2); SLE (3); MS (7); RA (63); GD (22); CLD (2); MG (2); PM (2); scleroderma (4); Addison disease (2); AIHA (2); PA (21); PBC (2)	Total (68445); IBD (193), CD (47), UC (146); SS (20); SLE (20); MS (72); RA (456); GD (82); CLD (9); MG (12); PM (7); scleroderma (5); Addison disease (14); AIHA (7); PA (89); PBC (7)	The Danish National Hospital Register	Matched 5 controls to each case based on year of birth and sex
Hsu et al. (20)	China	2000-2008	Retrospective cohort study	IBD (37/1698)	IBD (133/6792)	The National Health Research Institutes (NHRI)	Matched 4 controls to each case based on age and sex
Wu et al. (17)	China	2000-2010	Case-control study	SS (143/7716) SLE (22/7716)	SS (893/75129) SLE (173/75129)	Longitudinal Health Insurance Database (LHID) and National Health Institutes Research Database (NHIRD)	Matched 10 controls to each case based on age, gender and index date
Camacho-Soto et al. (21)	USA	2004-2009	Case-control study	IBD (2599/89790) CD (749) UC (1583)	IBD (2381/118095) CD (708) UC (1405)	Medicare base file (BASF)	Controls were a random sample of remaining beneficiaries
Bacelis et al. (22)	Sweden	1964-2016	Case-control study	RA (68/8256)	RA (1126/82452)	Socialstyrelsen (SOS), a Swedish governmental agency managing medical registries	Matched 10 controls to each case based on birth year, sex, birth location, and time of follow-up
Paakinaho et al. (23)	Finland	1996-2015	Case-control study	RA (315/1571)	NA	The Finnish Parkinson’s disease (FINPARK) cohort	Matched on age, sex, and region of residence
Cases group for autoimmune diseases							
Ludvigsson et al. (24)	Sweden	1964-2003	Retrospective cohort study	CLD (26/14345)	CLD (139/69958)	The Swedish National Board of Health and Welfare used the Swedish national inpatient register (IPR)	Matched on age, sex, calendar year and county
Taghipour et al. (25)	UK	2004-2008	Case-control study	BP (4/90)	BP (3/141)	Specialist outpatient center for immunobullous diseases at a teaching hospital in Oxford, England	Matched on age and sex
Bastuji-Garin et al. (26)	France	2003-2007	Case-control study	BP (28/201)	BP (20/345)	11 dermatology departments (11 hospitals) in France	Matched on age, gender, center, and place of residence
Chen et al. (27)	China	1997-2008	Case-control study	BP (416/3489)	BP (658/17425)	National Health Insurance Research Database (NHIRD) and Longitudinal Health Insurance Database 2000 (LHID 2000)	Matched 5 controls to each case based on age and sex
Langan et al. (28)	UK	1996-2006	Case-control study	BP (26/868)	BP (36/3453)	The Health Improvement Network database	Matched 4 controls to each case based on age and sex
Li et al. (12)	Sweden	1964-2007	Retrospective cohort study	CD (13/22750), UC (46/27881); SS (5/1360); SLE (8/5677); T1D (2/21946); MS (23/12503); RA (132/52994); GD (166/34735); BD (10/2718); CLD (6/10544); MG (7/2479); PM (2/1417); scleroderma (2/712); Addison disease (1/1841); AIHA (2/830); PA (56/5617); PBC (4/1379)	General population	The Primary Health Care Research Center-MigMed Database, Lund University	Sweden age and sex specific general population incidence rates for PD
Brick et al. (29)	USA	1960-2009	Case-control study Retrospective cohort study	BP (3/87) BP (4/84)	BP (1/261) BP (2/251)	Databases of the Rochester Epidemiology Project	Matched 3 controls to each case based on age and sex
Nielsen et al. (30)	Denmark	1977-2011	Prospective cohort study	MS (26/15557)	General population	Danish Multiple Sclerosis Registry (DMSR)	Corresponding national sex-, age- and period-specific incidence rates for pd in the Danish population
Teixeira et al. (31)	Portugal	1998-2010	Case-control study	BP (2/77)	BP (4/176)	Coimbra University Hospital	Matched 2 controls to each case based on age and sex
Klimek et al. (32)	Austria	2006-2007	Retrospective cohort study	T1D (N/16667)	T1D (N/1862258)	Database of the Main Association of Austrian Social Security Institutions	The total sample of inpatients
Liu et al. (33)	China	2000-2010	Prospective cohort study	SLE (55/12817)	SLE (393/51268)	The National Health Insurance Research Database (NHIRD)	Matched 2 controls to each case based on age and sex
Lin et al. (34)	China	2000-2011	Retrospective cohort study	IBD (106/8373) CD (97) UC (9)	IBD (290/33492)	Health Insurance Database 2000 (LHID 2000)	Matched 4 controls to each case based on age and sex
Sung et al. (2016) (35)	China	1998-2010	Retrospective cohort study	RA (360/33221)	RA (2381/132884)	Catastrophic Illness Patient Database (RCIPD)	Matched 4 controls to each case based on age and sex
Thormann et al. (36)	Denmark	1980-2005	Prospective cohort study	MS (34/8947)	MS (73/44733)	Danish Multiple Sclerosis Registry (DMSR)	Matched 5 controls to each case based on age, sex and municipality
Bählera et al. (37)	Switzerland	2014	Cross-sectional study	IBD (72/4197)	IBD (5184/1114638)	The Helsana Insurance Group	The helsana insurance group without IBD
Daneshpazhooh et al. (38)	Iran	2006-2011	Case-control study	BP (4/160)	BP (7/317)	Autoimmune Bullous Diseases Research Center, Razi Hospital, Tehran	Matched 2 controls to each case based on age and sex
Khosravani et al. (39)	Iran	2001-2016	Cross-sectional study	BP (3/87)	BP (4/184)	Faghihi Hospital, Shiraz, Iran	Matched 2 controls to each case based on age and sex
Kibsgaard et al. (40)	Denmark	1977-2015	Prospective cohort study	BP (78/3281)	BP (352/32213)	The Danish National Patient Registry (DNPR)	Matched 10 controls to each case based on age and sex
Sim et al. (41)	Singapore	2005-2014	Case-control study	BP (15/105)	BP (4/315)	Department of Dermatology, Singapore General Hospital	Matched 3 controls to each case based on age and sex
Yu Phuan et al. (42)	Singapore	2010-2015	Case-control study	BP (18/103)	BP (7/103)	Tan Tock Seng Hospital, Singapore	Matched on age and sex
Chang et al. (14)	China	2001-2012	Retrospective cohort study	SS (215/8422) SLE (49/3055) RA (379/19542)	SS (2285/138424) SLE (2285/138424) RA (2285/138424)	National Health Insurance Research Database (NHIRD)	Matched on age and sex
Jeon et al. (43)	South Korea	2006-2013	Case-control study	BP (6/103)	General population	Chonnam National University Hospital (CNUH) in Gwangju	Age-matched general population
Kridin et al. (44)	Israel	2004-2014	Cross-sectional study	BP (175/1985)	BP (437/9874)	Clalit Health Services (CHS) database	Matched 5 controls to each case based on sex, age, and ethnicity
Peter et al. (45)	USA	2000-2016	Retrospective cohort study	IBD (371/144018), CD (122/56507), UC (243/84436)	IBD (1425/720090) CD (480) UC (913)	The truven health marketscan commercial database and the medicare supplemental database	Matched 5 controls to each case based on age and sex
Villumsen et al. (15)	Denmark	1997-2014	Retrospective cohort study	IBD (335/76477)	IBD (39784/7548295)	The danish national patient register (NPR)	Matched on sex, age and vital status
Chen et al. (16)	China	2000-2012	Retrospective cohort study	SS (44/4053)	SS (13/4053)	The longitudinal health insurance database 2000 (LHID2000)	Matched 1 controls to each case based on age, sex and comorbidities
Ju et al. (46)	China	2000-2010	Retrospective cohort study	SS (159/12640)	SS (452/50560)	The National Health Insurance Research Database (NHIRD)	Matched 4 controls to each case based on age and sex
Papakonstantinou et al. (47)	Germany	2011-2015	Case-control study	BP (9/183)	BP (6/348)	Clinic in Germany	Matched on age and sex
Park et al. a (48)	South Korea	2010-2013	Retrospective cohort study	IBD (92/38861) CD (15/12631) UC (77/26230)	IBD (134/116583) CD (19/37893) UC (115/78690)	The national health insurance service (NHIS)	Matched 3 controls to each case based on age and sex
Park et al. b (49)	South Korea	2010-2013	Prospective cohort study	BD (50/11525)	BD (51/34575)	Korean national health insurance service (NHIS)	Matched 3 controls to each case based on age and sex
Weimers et al. (50)	Sweden	2002-2014	Prospective cohort study	IBD (103/39652) CD (23/11428) UC (69/24422)	IBD (1556/396520)	The swedish national patient register (NPR)	Matched 10 controls to each case based on sex, age, calendar year, and place of residence
Hsu et al. (51)	China	2000-2014	Retrospective cohort study	SS (273/17028)	SS (798/68094)	National Health Insurance (NHI) Research Database (NHIRD)	Matched 4 controls to each case based on gender, age group, and comorbidities
Noh et al. (52)	South Korea	2002-2006	Retrospective cohort study	IBD (25/411)	IBD (55/1405)	The nationwide administrative claims-based database of the national health insurance service (NHIS)	NA
Coates et al. (53)	USA	2005-2014	Retrospective cohort study	IBD (68/154051) CD (35) UC (33)	IBD (64/154051) CD (26) UC (38)	The marketscan commercial claims and encounters database	Matched 1 controls to each case based on age
Kridin et al. (2021) (54)	Germany	2008-2011	Cross-sectional study	BP (129/1743)	BP (292/10141)	Computerized data set of techniker krankenkasse	Matched 6 controls to each case based on age and sex
Kronzer et al. (55)	USA	2009-2020	Case-control study	RA (44/821)	RA (182/2455)	Mayo Clinic Biobank	Matched 3 controls to each case based on age, sex, recruitment year, and location
Sayar et al. (56)	Turkey	1987-2021	Case-control study	BP (3/145)	BP (5/310)	Department of Dermatology of the Istanbul Faculty of Medicine	Matched on age and sex
Cho et al. (57)	South Korea	2009-2014	Prospective cohort study	GD (301/65380)	GD (1097/326900)	National Health Information Database (NHID)	Matched 5 controls to each case based on age and sex
Kim et al. (58)	South Korea	2011-2017	Prospective cohort study	IBD (98/24830) CD (12/4454) UC (86/20376)	IBD (256/99320)	The National Health Insurance Service (NHIS)	Matched 4 controls to each case based on age and sex
Kwon et al. (59)	South Korea	2010-2017	Retrospective cohort study	MS (21/1380)	MS (14/6900)	The Korean National Health Insurance Service	Matched 5 controls to each case based on age, sex, hypertension, diabetes and dyslipidaemia

NA, not applicable.

The search identified clinical studies with 17 AIDs and PD. A total of 14,276,464 individuals, 873,643 patients, and 13,402,821 controls were included in the studies, including 752,488 patients with AIDs and 121,155 with PD. For the reported AIDs, Rugbjerg et al. (13) conducted a national case-control study in Denmark and reported 32 AIDs before diagnosis in 13,695 patients with PD. Li et al. (12) conducted a national epidemiological study in Sweden and reported a total of 33 AIDs in 310,522 patients, with 932 subsequently presenting with PD. In addition, 17 (37.0%) studies reported on BP, 12 (26.1%) on IBDs (including UC and CD), 7 (15.2%) on SS, 7 (15.2%) on RA, 4 (8.7%) on SLE, 5 (10.9%) on MS, 3 (6.5%) on CLD, 3 (6.5%) on GD, 2 (4.3%) on T1D, 2 (4.3%) on BD, 2 (4.3%) on MG, 2 (4.3%) on PM, 2 (4.3%) on scleroderma, 2 (4.3%) on Addison’s disease, 2 (4.3%) on AIHA, 2 (4.3%) on PA, and 2 (4.3%) on PBC. Six studies included patients with PD as the test group, and 39 used patients with AIDs as the test group. Most studies matched 1–10 controls per case based on age and sex. **Table 2** lists the studies included in the meta-analysis that included the correlation between PD and AIDs.

**Table 2:** Relationship between Parkinson’s disease and autoimmune disease.

Study	Measures reported	Risk estimates (in original reports)	Risk factors adjusted	Risk estimates (calculated/with correction)	Study Quality
Ludvigsson et al. (24)	HR	CLD: 1.20 (0.80-1.90)	NA	NA	8/9
Rugbjerg et al. (13)	OR	IBD: 1.35 (0.99-1.83); CD: 1.10 (0.50-2.10); UC: 1.30 (0.90-1.80); SS: 0.50 (0.12-2.14); SLE: 0.75 (0.22-2.52); MS: 0.49 (0.22-1.06); RA: 0.70 (0.53-0.90); GD: 1.34 (0.84-2.15); CLD: 1.11 (0.24-5.14)	NA	NA	7/9
Taghipour et al. (25)	OR	BP: 2.70 (0.60-11.60)	Age and sex	BP: 2.60 (0.60-11.40)	8/9
Bastuji- Garin et al. (26)	OR	BP: 2.16 (1.09-4.27)	NA	NA	7/9
Chen et al. (27)	OR	BP: 3.45 (3.03-3.92)	Age, sex, follow-up time, Charlson score and healthcare utilization	BP: 3.49 (3.05-3.98)	8/9
Langan et al. (28)	OR	BP: 3.00 (1.80-5.00)	Charlson scores not including neurological conditions	BP: 2.90 (1.70-4.90)	9/9
Li et al. (12)	SIR	NA	Age, period, socioeconomic status, region of residence, hospitalization of COPD, and alcoholism and alcohol-related liver disease	CD: 0.62 (0.33-1.07); UC: 1.23 (0.90-1.64); SS: 2.01 (0.63-4.72); SLE: 1.00 (0.43-1.97); MS: 1.66 (1.05-2.50); RA: 1.07 (0.89-1.26); GD: 1.63 (1.39-1.90); CLD: 1.01 (0.36-2.21)	7/9
Brick et al. (29)	OR	BP: 9.00 (0.94-86.52)	NA	NA	8/9
	HR	BP: 8.56 (1.55-47.25)	NA	NA	8/9
Teixeira et al. (31)	OR	BP: 4.91 (0.88-27.44)	NA	NA	6/9
Liu et al. (33)	HR	SLE: 0.60 (0.45-0.79)	Age and comorbidities	SLE: 0.68 (0.51-0.90)	9/9
Lin et al. (34)	HR	IBD: 1.43 (1.15-1.79); CD: 1.45 (1.15-1.83); UC: 1.25 (0.64-2.42)	Age, sex, and comorbidities	IBD: 1.35 (1.08-1.68); CD: 1.40 (1.11-1.77); UC: 0.94 (0.49-1.84)	9/9
Sung et al. (35)	HR	RA: 0.62 (0.55-0.69)	Age and comorbidities, and nonsteroidal anti- inflammatory drugs use	RA: 0.65 (0.58-0.73)	9/9
Thormann et al. (36)	HR	MS: 2.50 (1.66-3.76)	NA	NA	7/9
Bählera et al. (37)	OR	NA	Age, sex, language area, type of insurance coverage, and urbanization	IBD: 0.92 (0.67-1.27)	7/9
Daneshpazhooh et al. (38)	OR	BP: 1.14 (0.33-3.94)	NA	NA	7/9
Khosravani et al. (39)	OR	BP: 1.61 (0.35-7.34)	NA	NA	6/9
Kibsgaard et al. (40)	RR	BP: 2.18 (1.71-2.77)	NA	NA	7/9
Sim et al. (41)	OR	BP: 20.59 (4.69-90.49)	NA	NA	7/9
Wu et al. (17)	OR	SS: 1.56 (1.30-1.86); SLE: 1.24 (0.79-1.93)	NA	SS: 1.37 (1.15-1.65)	7/9
Yu Phuan et al. (42)	OR	BP: 2.90 (1.16-7.29)	Age, gender, race, functional status and any prescribed relevant neurological medications	BP: 2.13 (0.80-5.69)	9/9
Camacho-Soto et al. (21)	OR	NA	Age, race, sex, and probability of smoking, comorbidities	IBD: 0.85 (0.80-0.91); CD: 0.83 (0.74-0.93); UC: 0.88 (0.82-0.96)	7/9
Chang et al. (14)	HR	NA	Age group, sex, and comorbidities	SS: 1.56 (1.35-1.79); RA: 1.14 (1.03-1.28)	9/9
Jeon et al. (43)	OR	BP: 3.45 (1.49-7.98)	NA	NA	6/9
Kridin et al. (44)	OR	BP: 2.09 (1.74-2.51)	Charlson Comorbidity Index score	1.97 (1.64-2.36)	8/9
Peter et al. (45)	IR	IBD: 1.28 (1.14-1.44); CD: 1.26 (1.03-1.54); UC: 1.30 (1.13-1.50)	Time-varying age group and sex, and offset by time	IBD: 1.28 (1.14-1.44); CD: 1.26 (1.03-1.53); UC: 1.31 (1.14-1.51)	9/9
Villumsen et al. (15)	HR	IBD: 1.24 (1.12-1.38)	Gender and age; comorbidity index	IBD: 1.22 (1.09-1.35); CD: 1.35 (1.20-1.52); UC: 1.12 (0.89-1.40)	9/9
Chen et al. (16)	RR	NA	Age and sex	SS: 3.39 (1.83-6.27)	8/9
Papakonstantinou et al. (47)	OR	BP: 2.9 (1.00-8.40)	NA	NA	7/9
Park et al. (48) a	HR	NA	Age, sex, place of residence, income level, and comorbidities	IBD: 1.87 (1.43-2.44); CD: 2.23 (1.12-4.45); UC: 1.85 (1.38-2.48)	9/9
Weimers et al. (50)	HR	NA	Sex, age, index date, and place of residency	IBD: 1.30 (1.00-1.60); CD: 1.10 (0.70-1.70); UC: 1.30 (1.00-1.70)	9/9
Bacelis et al. (22)	OR	RA: 0.60 (0.46-0.77)	NA	NA	8/9
Coates et al. (2021)	HR	NA	Age, sex, residence, region, smoking alcohol consumption and comorbidities	IBD: 1.01 (0.72-1.42); CD: 1.33 (0.80-2.21); UC: 0.81 (0.51-1.29)	9/9
Kridin et al. (54)	OR	BP: 2.71 (2.19-3.35)	NA	NA	7/9
Kronzer et al. (55)	OR	RA: 0.71 (0.50-0.99)	Age, sex, race, BMI, education, smoking	RA: 0.70 (0.49-0.98)	8/9
Sayar et al. (56)	OR	BP: 1.29 (0.30-5.47)	NA	NA	7/9
Cho et al. (57)	HR	GD: 1.37 (1.21-1.56)	Age, sex, household income, and comorbidities	GD:1.33 (1.17-1.51)	7/9
Kim et al. (58)	HR	IBD: 1.55 (1.23-1.96); CD: 1.05 (0.59-1.88); UC: 1.66 (1.30-2.12)	Age, sex, residential area, and comorbidities	IBD: 1.56 (1.24-1.97); CD: 1.03 (0.58-1.84); UC: 1.69 (1.32-2.15)	9/9
Kwon et al. (59)	HR	NA	Age, sex and comorbidities	MS: 7.73 (3.87-15.47)	9/9

NA, not applicable.

### 3.3 Excluded studies

The number of clinical studies on T1D, BD, MG, PM, scleroderma, Addison’s disease, AIHA, PA, and PBC with PD was <3; therefore, those diseases were not included in the meta-analysis. **Supplementary Table 2** lists the studies that were not included in the meta-analysis. Among them, results from Klimek et al.’s study (32) suggested a significantly higher risk of PD combined with T1D (relative risk [RR]=2.30, 95% CI: 1.90–2.70). Results from Park et al.’s study (49) suggested a significantly higher risk of PD combined with BD of 2.47 (1.65–3.68). Rugbjerg et al. (13) and Li et al. (12) included studies of multiple AIDs and risk of PD; however, a meta-analysis was not performed for some of these diseases due to the small number of studies that could corroborate any associated findings.

Both Hsu et al.’s (20) and Lin et al.’s (34) studies are reports on IBD with patients originating from the same database; therefore, the study with the larger sample size (i.e., Lin et al.’s study) was selected, excluding that by Hsu et al. (20). Studies by Ju et al. (46), Hsu et al. (51), and Chang et al. (14) all originate from a study on SS, with patients from the same database. The study with the larger sample size (i.e., Chang et al.’s study) was selected synthetically, excluding those by Ju et al. (46) and Hsu et al. (51). Studies by Nielsen et al. (30) and Thormann et al. (36) are Danish reports on MS with patients derived from the same database; because of which, Thormann et al.’s study (36) was selected as it included more complete information, excluding Nielsen et al.’s report (30). A retrospective cohort study in South Korea by Noh et al. (52) compared conventional treatment and combination treatment for IBD and found a reduced risk of PD in the combined treatment group without normal controls, because of which it was excluded. Paakinaho et al.’s study (23) examined the association between RA and PD by improving anti-rheumatic drugs, which did not match the data of this study; thus, it was excluded from the meta-analysis. Thirty-seven studies were eventually included in the meta-analysis.

### 3.4 Quality of evidence

The NOS scores of the included studies ranged from 6 to 9, indicating a high level of overall quality. The studies had clear definitions of exposure and outcome, appropriate adjustment for confounders, and sufficiently long follow-up (**Supplementary Table 3**).

### 3.5 Meta-analysis

**Figure 2:**
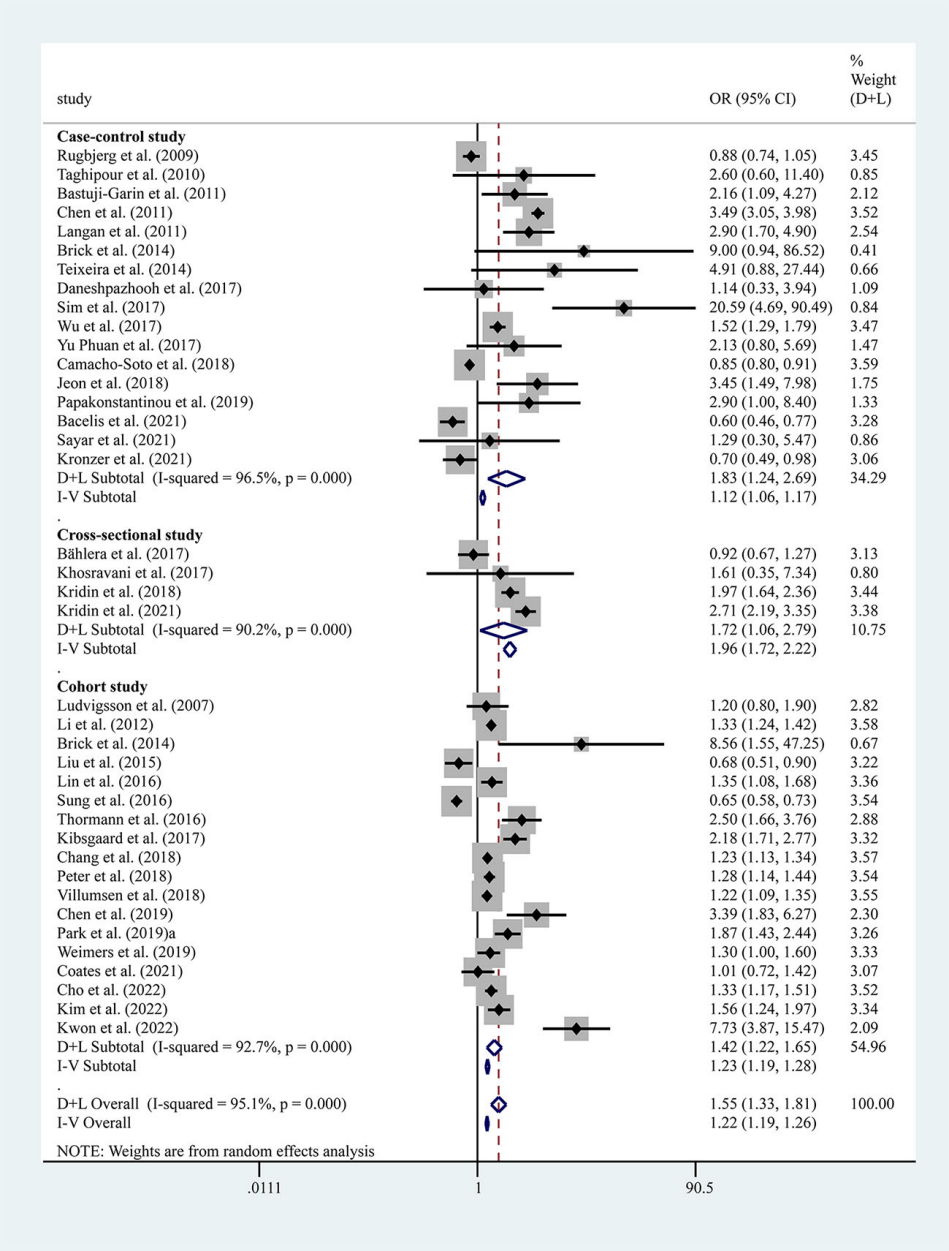
Forest plots of studies association between PD and AIDs. The size of the square is proportional to study-specific statistical weights, horizontal lines represent 95% confidence interval and diamonds represent summary measures of association.

The results of the meta-analysis of 17 case-control, 4 cross-sectional, and 18 cohort studies showed a significantly higher overall risk of PD with AIDs (OR=1.55, 95% CI: 1.33–1.81, P=0.000, I^2 =^ 95.1%, large heterogeneity), with case-control (OR=1.83, 95% CI: 1.24–2.69, P=0.000, I^2 =^ 96.5%, large heterogeneity), cohort (OR=1.42, 95% CI: 1.22–1.65, P=0.000, I^2 =^ 92.7%, large heterogeneity) and cross-sectional studies (OR= 1.72, 95% CI: 1.06–2.79, P=0.000, I^2 =^ 90.2%, large heterogeneity) all suggesting an increased risk of PD with AIDs (**Figure 2**). The findings suggested high heterogeneity, and a subgroup analysis was performed to find the source of heterogeneity. However, we found no significant differences in risk by study type, gender, age, race, and study design (**Table 3**). Therefore, we performed separate analyses to determine the relationship between different types of AID and PD.

**Table 3:** Analyses of subgroups relating autoimmune diseases to Parkinson’s disease.

Factor	No. of studies	OR (95%CI)	I^2^ (%)	P	Model
All autoimmune diseases	39	1.55 (1.33-1.81)	95.1	0.000	R
Study type					
Case-control study	17	1.83 (1.24-2.69)	96.5	0.000	R
Cross-sectional study	4	1.72 (1.06-2.79)	90.2	0.000	R
Cohort study	18	1.42 (1.22-1.65)	92.7	0.000	R
Gender					
Male	13	1.51 (1.11-2.05)	95.2	0.000	R
Female	13	1.44 (1.13-1.82)	95.7	0.000	R
Age					
< 65 years old	7	1.02 (0.73-1.43)	93.9	0.000	R
≥ 65 years old	7	1.06 (0.82-1.36)	92.7	0.000	R
Race					
Europ	22	1.36 (1.15-1.62)	92.6	0.000	R
Asia	17	1.82 (1.37-2.43)	96.5	0.000	R
Study design					
Prospective	6	1.45 (1.08-1.94)	89.4	0.000	R
Retrospective	33	1.58 (1.33-1.89)	95.6	0.000	R
Types of autoimmune diseases					
BP	17	2.67 (2.15-3.31)	63.3	0.000	R
IBD	9	1.30 (1.18-1.45)	54.6	0.024	R
CD	9	1.30 (1.20-1.42)	25.2	0.220	F
UC	9	1.31 (1.14-1.50)	51.7	0.035	R
SS	5	1.61 (1.24-2.09)	61.9	0.033	R
SLE	4	0.82 (0.66-1.03)	42.8	0.155	F
MS	4	2.02 (0.87-4.70)	89.5	0.001	R
RA	6	0.79 (0.61-1.03)	92.4	0.000	R
GD	3	1.45 (1.24-1.70)	50.0	0.136	R
CLD	3	1.16 (0.79-1.69)	0.0	0.944	F

R, Random model; F, Fixed model.

**Figure 3:**
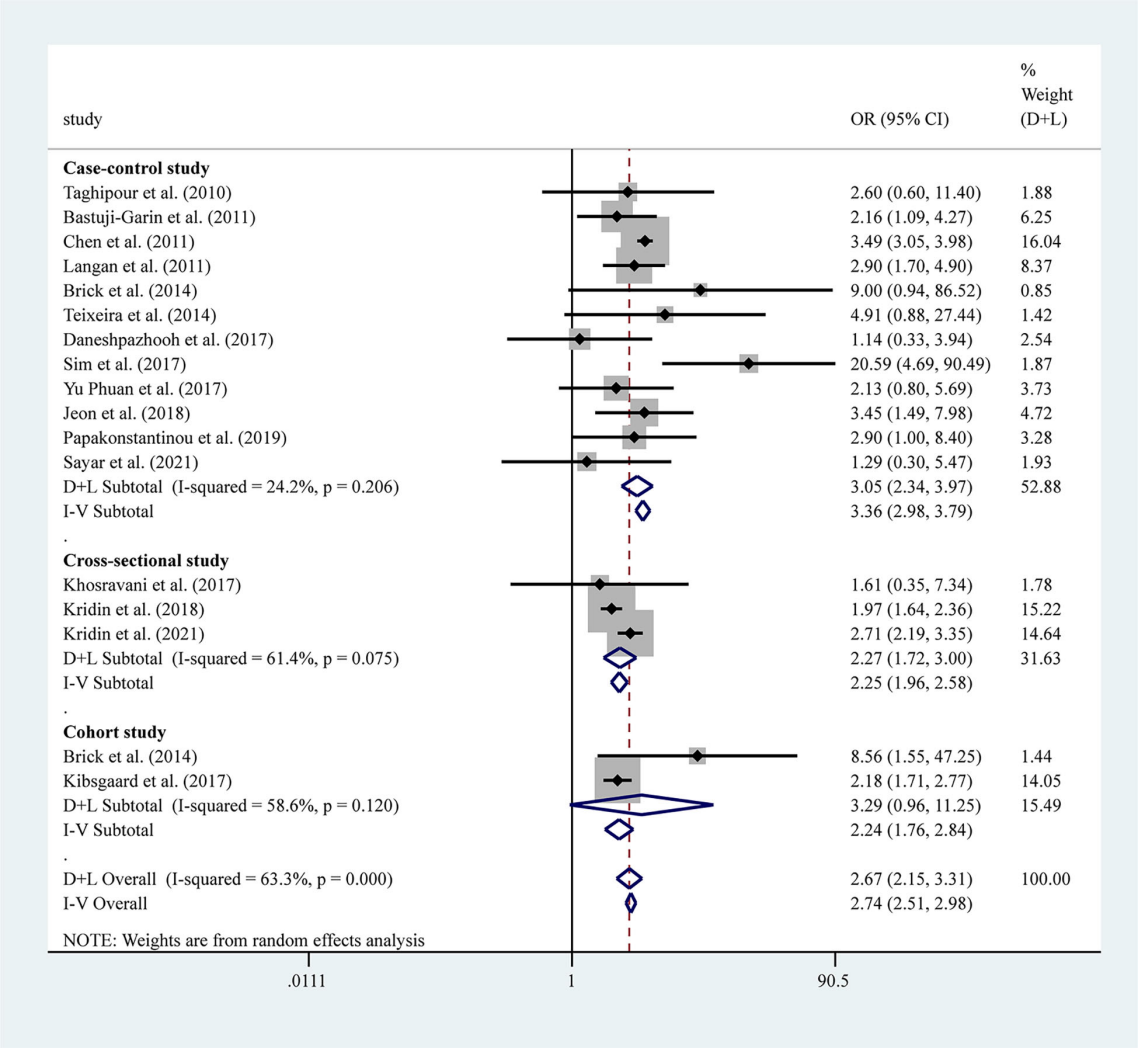
Forest plots of studies association between PD and BP.

Seventeen studies reported the risk between BP and PD, and the results showed a significantly higher risk for PD combined with BP (OR=2.67, 95% CI: 2.15–3.31, P=0.000, I^2 =^ 63.3%, moderate heterogeneity). According to the grouping of study types, the results showed no heterogeneity in the effect values of the 12 case-control studies combined (OR=3.36, 95% CI: 2.98–3.79, P=0.206, I^2 =^ 24.4%) (**Figure 3**).

**Figure 4:**
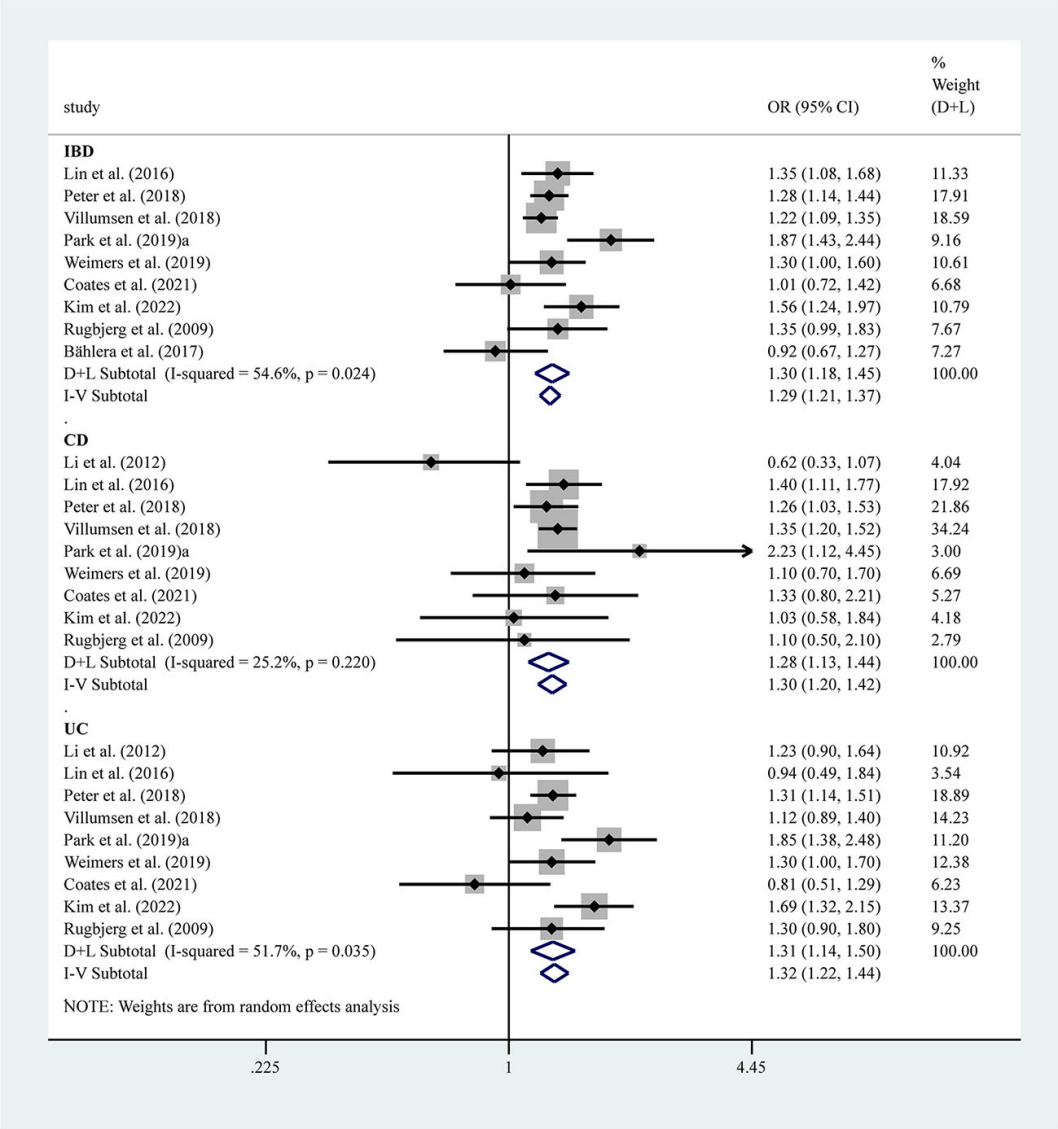
Forest plots of studies association between PD and IBD (including UC and CD).

Ten studies reported the risk between IBD and PD and showed a significantly higher risk of PD combined with IBD (OR=1.24, 95% CI: 1.04–1.47, P=0.001, I^2 =^ 91.0%), with large heterogeneity. However, when Camacho-Soto et al.’s study (21) was removed from this analysis, heterogeneity was significantly lower after combining effect values and the association between PD and IBD was stronger. We therefore included nine studies, excluding Camacho-Soto et al.’s report (21), which showed a significantly higher risk of PD with IBD (OR=1.30, 95% CI: 1.18–1.45, P=0.024, I^2 =^ 54.6, moderate heterogeneity). Subtypes of IBD, UC (OR=1.31, 95% CI: 1.14–1.50, P=0.035, I^2 =^ 51.7%, moderate heterogeneity), and CD (OR=1.30, 95% CI: 1.20–1.42, P=0.220, I^2 =^ 25.2%, mild heterogeneity) were also associated with a significantly higher risk of PD (**Figure 4**).

**Figure 5:**
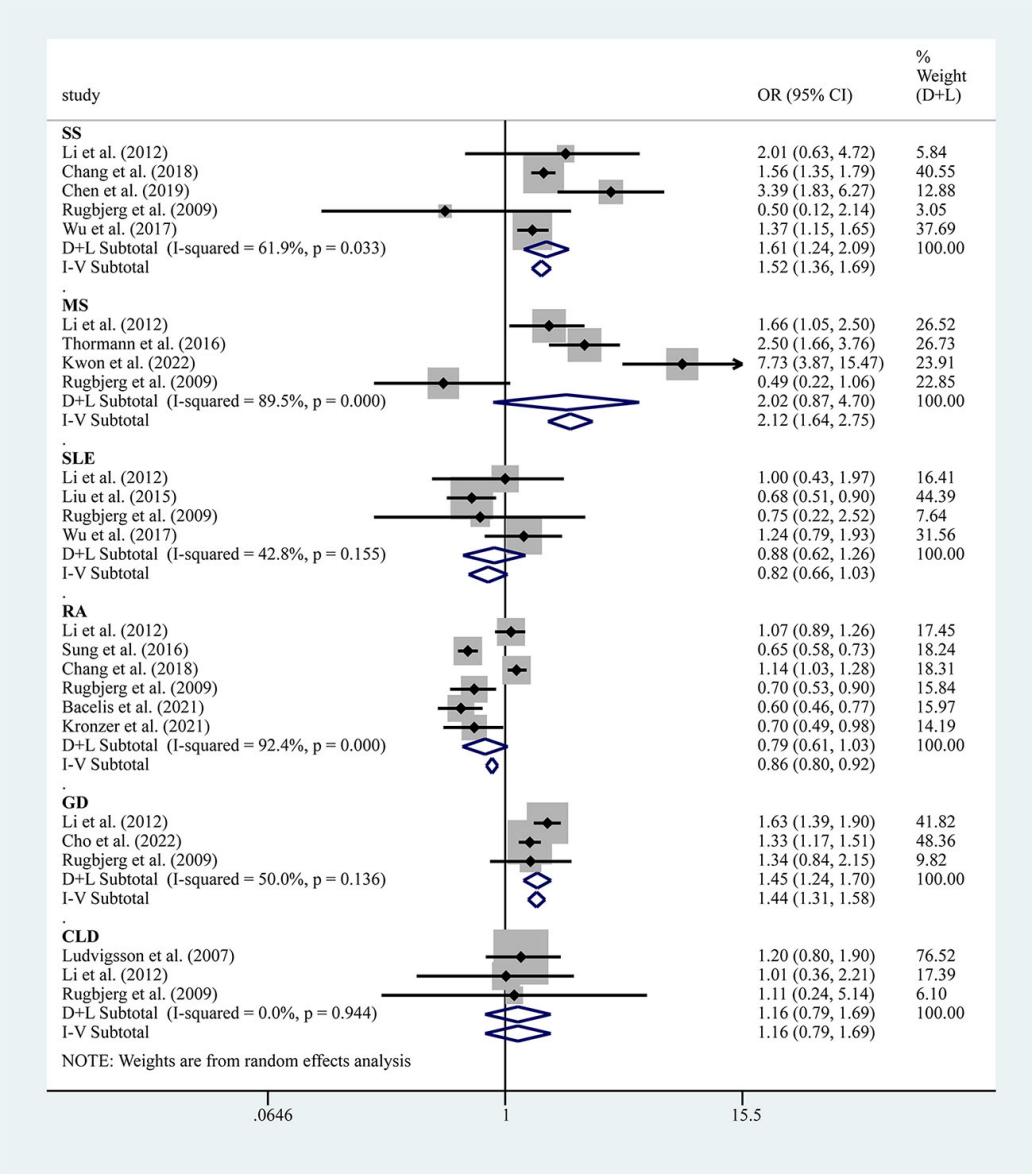
Forest plots of studies association between PD and AIDs (including SS, SLE, MS, RA, GD and CLD).

Five studies reported the risk between SS and PD, and the results showed an increased risk of PD combined with SS (OR=1.61, 95% CI: 1.24–2.09, P=0.033, I^2 =^ 61.9%, moderate heterogeneity) (**Figure 5**). However, sensitivity analysis showed fewer stable results, and two studies, i.e., those by Chang et al. (14) and Wu et al. (17), would have a greater impact on the results (**Supplementary Table 4**, **Supplementary Figure 1F**). Three studies reported the risk between GD and PD, and the results showed an increased risk of PD combined with GD (OR=1.45, 95% CI: 1.24–1.70, P=0.136, I^2 =^ 50.0%, mild heterogeneity) (**Figure 5**).

Six studies reported the risk between RA and PD, and the results showed that the risk of PD combined with RA was not significant (OR=0.79, 95% CI: 0.61–1.03, P=0.000, I^2 =^ 92.4%, large heterogeneity) (**Figure 5**). Notably, after removing this study by Chang et al. (14) from the sensitivity analysis, the combined effect values became meaningful, showing a negative association between PD and RA (**Supplementary Figure 1I**).

Four studies reported the risk between SLE and PD, and the results showed that the risk for PD combined with SLE was not significant (OR=0.82, 95% CI: 0.66–1.03, P=0.155, I^2 =^ 42.8%, large heterogeneity) (**Figure 5**). Four studies reported the risk between MS and PD, and the results showed that the risk of PD combined with MS was not significant (OR=2.02, 95% CI: 0.87–4.70, P=0.001, I^2 =^ 89.5%, large heterogeneity) (**Figure 5**). Three studies reported the risk between CLD and PD and showed that the risk for PD combined with CLD was not significant (OR=1.16, 95% CI: 0.79–1.69, P=0.944, I^2 =^ 0.0%, no heterogeneity) (**Figure 5**).

### 3.6 Publication bias

The P-values for both Begg’s and Egger’s tests were >0.05, indicating a low likelihood of potential publication bias (**Supplementary Table 5**).

### 3.7 Sensitivity analysis

Sensitivity analyses removed each included study individually and performed a pooled analysis of the remaining studies to assess whether the individual included reports had a greater impact on the results of the overall meta-analysis. Of the several analyses with positive results, the remaining studies analyzed did not have a disproportionate effect on the results of the meta-analysis, except for the less stable results of SS, indicating that the results of the remaining studies were stable and reliable (**Supplementary Table 4**, **Supplementary Figure 1**).

## 4 Discussion

In this meta-analysis of 38 population-based cohort, case-control, and cross-sectional studies, PD may be associated with multiple AIDs, including BP, IBD, CD, UC, SS, and GD, but may not be associated with MS, SLE, RA, and CLD. To the best of our knowledge, this study is the first to comprehensively synthesize the available population-based research evidence on the relationship between PD and AIDs.

Our study benefited from a comprehensive search strategy that included 34 common AIDs, essentially encompassing the majority of reported clinical studies on the relationship between AIDs and PD. The final pooled inclusion of more than 10 million subjects from different geographic regions, including clinical studies in 16 countries with populations in Asia, Europe, and North America, provides reliable evidence of the relationship between AIDs and PD from large-scale subject data. However, it is difficult to distinguish the sequence of development of PD and AIDs in multiple studies, and this study only analyzed the risk of PD combined with AIDs to demonstrate whether there is a correlation between the two, not to determine the causal relationship.

The increased risk of PD combined with AIDs may have a similar pathogenesis. Indeed, there is growing evidence that immune dysfunction is involved in the pathogenesis of PD (7, 60). Some studies have found that an aberrant immune response may start years before the diagnosis of PD (61). Sustained inflammatory response, T-cell infiltration, and glial cell activation play a crucial role in the degeneration of dopaminergic neurons (62, 63). Current experimental and genetic studies linking AIDs to PD have found a role for intestinal microflora, immune response, and genetic variants, although the mechanisms between PD and AIDs remain unclear (9, 10, 60, 63). In the following paragraphs we will discuss each of the several AIDs associated with PD.

A meta-analysis summarizing the association of BP with neurological disorders included eight studies on PD with BP, and the results suggested that patients with BP were more than three times more likely to have PD (RR=3.42, 95% CI: 3.01-3.87) (64). In contrast, our study included 17 papers on BP and PD, and the results suggested a 2.67-fold risk for BP combined with PD. Some studies have shown that BP is also associated with other neurological disorders, such as dementia, MS, epilepsy, stroke, and schizophrenia (47, 54, 64). Studies have shown that human skin and the brain contain BP180 antigen and BP230 antigen, and the mechanism may be that neurological disorders expose antigens such as BP180 and BP230 to the immune system and trigger a subsequent immune response that leads to the manifestation of BP (65, 66). Studies have shown that circulating IgG autoantibodies against BP180 are found in patients with Parkinson’s disease, but their significance for the development of BP is currently unknown, as these anti-BP180 antibodies neither bind to the basement membrane of the skin nor cause BP-like symptoms (67)..

Several clinical studies have shown that IBD, including CD and UC, is associated with an increased risk of PD (34, 45, 48), and a meta-analysis also showed an increased risk of IBD combined with PD (RR=1.24, 95% CI: 1.15-1.34) (63), which is consistent with our results. Gastrointestinal inflammation and neuroinflammation may be important causes of PD due to disorders of the gut-brain axis (68). It is believed that gastrointestinal inflammation promotes misfolding of alpha-synuclein, leading to its aggregation and prion-like propagation in the brain (69, 70). While IBD is a typical gastrointestinal disorder, pro-inflammatory immune response and homeostatic imbalance in the gut have an important role in the pathogenic process of IBD. Therefore, IBD may be involved in the disruption of the gut-brain axis through mechanisms, such as intestinal inflammation. The gut-brain axis may be an important link between PD and IBD. It has also been demonstrated that IBD and PD share common genetic risk profiles, such as *CARD15*, *LRRK2*, *HLA*, and *MAPT* genes (71, 72). Notably, a two-sample Mendelian randomization study on IBD and PD genetically predicted that neither IBD nor its subtypes CD and UC were associated with an increased risk of PD (73), although another Mendelian randomization study confirmed a causal relationship between PD and IBD (74).

The increased risk of SS combined with PD may be due to the role played by autoantibodies. We know that SS is caused by an immune-mediated mechanism, and autoantibodies (e.g., anti-SSA and anti-SSB), which are associated with central nervous system disorders, are commonly detected in patients with SS (75). The immune-mediated mechanism between SS and PD is postulated, and antibodies in patients with SS may damage the basal ganglia and cause PD. In patients with SS combined with PD, serum anti-β2 glycoprotein antibodies are strongly positive, and it is postulated that this antibody binds to antigen to form an immune complex that is deposited in the vessel wall, causing vasculitis and the clinical manifestations of PD (76).

Studies have shown that thyroid dysfunction (e.g., hyperthyroidism and hypothyroidism) can increase the risk of PD (77). Thyroid dysfunction affects oxidative stress, which contributes significantly to the loss of dopamine neurons and the progression of PD (78, 79). And it is possible that GD combined with PD may also be a result of the presence of common risk factors, such as vitamin D deficiency (80, 81).

In our study, the risk of PD combined with RA was not statistically significant. In contrast, another meta-analysis suggested a negative correlation between RA and PD (RR=0.74, 95% CI: 0.56–0.98) (82). A Mendelian randomization study also supported the protective effect of RA on PD (83). Several clinical studies have suggested a protective mechanism of RA against PD (22, 35, 55). Drugs commonly used by patients with RA are non-steroidal anti-inflammatory drugs (NSAIDs) and immunosuppressive drugs, and epidemiological studies suggest that regular use of anti-inflammatory drugs may be associated with a reduced risk of PD (84, 85). Therefore, it is also necessary to exclude the interference of NSAIDs when studying the correlation between PD and RA.

We did not find an association between PD and SLE, MS, or CLD. Notably, all three clinical studies of CLD included suggested no statistically significant association between PD and CLD. Nine AIDs, T1D, BD, MG, PM, scleroderma, Addison’s disease, AIHA, PA, and PBC, were also retrieved in this study for their association with PD but were not included in the meta-analysis due to an insufficient number of clinical studies. Clinical studies on these diseases also suggest that most are not statistically significantly associated with PD.

## 5 Limitations and future prospective

The sequential order of disease progression in PD and AIDs and potential causal relationship were difficult to determine in this study. In cross-sectional and case-control studies, the temporal relationship between the two conditions could not be resolved because the timing of diagnosis of the included study diseases was unclear. Moreover, alpha-synuclein can be detected in the gut and olfactory epithelium of PD patients many years ago, and this underlying pathological process takes place over about 20 years, with a significant loss of dopamine neurons in the brain by the time overt motor symptoms appear (86, 87). In addition, some AIDs such as SLE and RA may also go undetected for many years (88, 89), making it challenging to determine the exact time of disease onset to determine the sequence between the two. Most of the studies included in this study were retrospective observational studies with information derived from questionnaires, disease databases, or data from inpatient registries, and differences in diagnostic criteria for PD or AIDs may have biased the results of this meta-analysis. Most of the included studies were adjusted only for age and sex confounders, although they ignored other confounders such as potential risk factors for PD (tobacco, coffee, NSAIDs, and comorbidities, among others), which may limit the prevalence of AIDs with PD. Another limitation of this study is the moderate to high heterogeneity in most of the results, which may be attributed to differences in the study area, sample source, and study design, and we attempted to address the large heterogeneity by using a random effects model. In addition, some of the AIDs included in this study have fewer reported clinical studies and the association of these disorders with PD cannot be well assessed. In conclusion, due to some of the aforementioned limitations of this study, the final results obtained may not be very adequate.

To assess the relationship more accurately between PD and AIDs, we suggest that a prospective cohort study approach be used in the future. More accurate evidence of the relationship between PD and AIDs can be obtained by following up long enough to include a large sample size of the study population, using standardized hospital-based records of disease diagnosis, carefully selecting normal controls, and adjusting for potential confounders. Mechanistic studies on PD and AID comorbidity can improve our understanding of the pathogenesis of both diseases and, if a common pathogenic origin can be observed, help identify new therapeutic and diagnostic targets.

## 6 Conclusion

This meta-analysis provides evidence that patients with PD have a significantly increased risk for comorbid AIDs. However, for different AIDs, the OR varied widely, with BP showing the strongest association. Clinicians need to be aware of the potential coexistence of PD and AIDs when they are diagnosed. Further studies are needed to explore the potential molecular mechanisms underlying the interaction between PD and AIDs.

## Data availability statement

The original contributions presented in the study are included in the article/**Supplementary Material**. Further inquiries can be directed to the corresponding author.

## Author contributions

ML and BT are responsible for the study concept and design; ML, JW, and ZX are responsible for the data collection, data analysis, and interpretation; ML drafted the paper; BT supervised the study. All authors contributed to the article and approved the submitted version.
