# Supplementary material for: The association between Parkinson’s disease and autoimmune diseases: A systematic review and meta-analysis
Source: Front Immunol. 2023 Jan 25;14:1103053. doi: 10.3389/fimmu.2023.1103053 (PMC9905134; doi:10.3389/fimmu.2023.1103053)
Supplement: Supplementary file 1 [file DataSheet_1.docx]

**Supplementary Material**

**Table 1 (Page 2-9).** Search strategy in the PubMed database.

**Table 2 (page 9-12).** Quality assessment.

Study quality assessment using Newcastle-Ottawa scale for cohort studies (part 1).

Study quality assessment using Newcastle-Ottawa scale for case-control studies (part 2).

**Table 3 (page 13).** Correlation of Parkinson's disease with autoimmune disease in the excluded literature.

**Table 4 (page 14).** Results of Begg’s test and Egger’s test.

**Table 5 (page 15).** Sensitivity analysis, the impact of individual studies on the overall pooled result was assessed by excluding one study at a time.

**Figure 1 (page 16-21).** The effects of the individual studies on the pooled effect size of PD and AIDs risk.

**Table 1.** Search strategy in the PubMed database.

| 1. ((((((((((((Parkinson disease[MeSH Terms]) OR (Parkinson disease[Title/Abstract])) OR (Idiopathic Parkinson's Disease[Title/Abstract])) OR (Lewy Body Parkinson's Disease[Title/Abstract])) OR (Parkinson's Disease, Idiopathic[Title/Abstract])) OR (Parkinson's Disease, Lewy Body[Title/Abstract])) OR (Parkinson Disease, Idiopathic[Title/Abstract])) OR (Parkinson's Disease[Title/Abstract])) OR (Idiopathic Parkinson Disease[Title/Abstract])) OR (Lewy Body Parkinson Disease[Title/Abstract])) OR (Primary Parkinsonism[Title/Abstract])) OR (Parkinsonism, Primary[Title/Abstract])) OR (Paralysis Agitans[Title/Abstract]) |
| --- |
| 2. ((((Autoimmune Diseases[MeSH Terms]) OR (Autoimmune Diseases[Title/Abstract])) OR (Disease, Autoimmune[Title/Abstract])) OR (Diseases, Autoimmune[Title/Abstract])) OR (Autoimmune Disease[Title/Abstract]) |
| 3. ((((((((((((((((Polyradiculoneuropathy, Chronic Inflammatory Demyelinating[MeSH Terms]) OR (Polyradiculoneuropathy, Chronic Inflammatory Demyelinating[Title/Abstract])) OR (Polyneuropathy, Inflammatory Demyelinating, Chronic[Title/Abstract])) OR (Inflammatory Polyradiculopathy, Chronic[Title/Abstract])) OR (Chronic Inflammatory Polyradiculopathies[Title/Abstract])) OR (Chronic Inflammatory Polyradiculopathy[Title/Abstract])) OR (Inflammatory Polyradiculopathies, Chronic[Title/Abstract])) OR (Polyradiculopathies, Chronic Inflammatory[Title/Abstract])) OR (Polyradiculopathy, Chronic Inflammatory[Title/Abstract])) OR (Polyradiculoneuropathy, Chronic Inflammatory[Title/Abstract])) OR (Chronic Inflammatory Polyradiculoneuropathy[Title/Abstract])) OR (Chronic Inflammatory Polyradiculoneuropathies[Title/Abstract])) OR (Inflammatory Polyradiculoneuropathies, Chronic[Title/Abstract])) OR (Inflammatory Polyradiculoneuropathy, Chronic[Title/Abstract])) OR (Polyradiculoneuropathies, Chronic Inflammatory[Title/Abstract])) OR (Chronic Inflammatory Demyelinating Polyradiculoneuropathy[Title/Abstract])) OR (CIDP[Title/Abstract]) |
| 4. ((((((((((((((((((((((((((((((((((((((((((((((((((Guillain-Barre Syndrome[MeSH Terms]) ) OR (Guillain-Barre Syndrome[Title/Abstract])) OR (Guillain Barre Syndrome[Title/Abstract])) OR (Syndrome, Guillain-Barre[Title/Abstract])) OR (Guillaine-Barre Syndrome[Title/Abstract])) OR (Guillaine Barre Syndrome[Title/Abstract])) OR (Syndrome, Guillaine-Barre[Title/Abstract])) OR (Guillain-Barré Syndrome[Title/Abstract])) OR (Guillain Barré Syndrome[Title/Abstract])) OR (Guillain-Barré Syndromes[Title/Abstract])) OR (Syndrome, Guillain-Barré[Title/Abstract])) OR (Syndromes, Guillain-Barré[Title/Abstract])) OR (Landry-Guillain-Barre Syndrome[Title/Abstract])) OR (Landry Guillain Barre Syndrome[Title/Abstract])) OR (Syndrome, Landry-Guillain-Barre[Title/Abstract])) OR (Acute Autoimmune Neuropathy[Title/Abstract])) OR (Acute Autoimmune Neuropathies[Title/Abstract])) OR (Autoimmune Neuropathies, Acute[Title/Abstract])) OR (Autoimmune Neuropathy, Acute[Title/Abstract])) OR (Neuropathies, Acute Autoimmune[Title/Abstract])) OR (Neuropathy, Acute Autoimmune[Title/Abstract])) OR (Acute Infectious Polyneuritis[Title/Abstract])) OR (Infectious Polyneuritis, Acute[Title/Abstract])) OR (Polyneuritis, Acute Infectious[Title/Abstract])) OR (Guillain-Barre Syndrome, Familial[Title/Abstract])) OR (Familial Guillain-Barre Syndrome[Title/Abstract])) OR (Familial Guillain-Barre Syndromes[Title/Abstract])) OR (Guillain Barre Syndrome, Familial[Title/Abstract])) OR (Guillain-Barre Syndromes, Familial[Title/Abstract])) OR (Syndrome, Familial Guillain-Barre[Title/Abstract])) OR (Syndromes, Familial Guillain-Barre[Title/Abstract])) OR (Acute Inflammatory Demyelinating Polyneuropathy[Title/Abstract])) OR (Inflammatory Polyneuropathy Acute[Title/Abstract])) OR (Polyneuropathy Acute, Inflammatory[Title/Abstract])) OR (Acute Inflammatory Demyelinating Polyradiculoneuropathy[Title/Abstract])) OR (Acute Inflammatory Polyneuropathy[Title/Abstract])) OR (Acute Inflammatory Polyneuropathies[Title/Abstract])) OR (Inflammatory Polyneuropathies, Acute[Title/Abstract])) OR (Inflammatory Polyneuropathy, Acute[Title/Abstract])) OR (Polyneuropathies, Acute Inflammatory[Title/Abstract])) OR (Polyradiculoneuropathy, Acute Inflammatory[Title/Abstract])) OR (Acute Inflammatory Polyradiculoneuropathies[Title/Abstract])) OR (Inflammatory Polyradiculoneuropathies, Acute[Title/Abstract])) OR (Polyradiculoneuropathies, Acute Inflammatory[Title/Abstract])) OR (Polyneuropathy, Inflammatory Demyelinating, Acute[Title/Abstract])) OR (Demyelinating Polyradiculoneuropathy, Acute Inflammatory[Title/Abstract])) OR (Polyneuropathy, Acute Inflammatory[Title/Abstract])) OR (Polyradiculoneuropathy, Acute Inflammatory Demyelinating[Title/Abstract])) OR (Inflammatory Demyelinating Polyradiculoneuropathy, Acute[Title/Abstract])) OR (Acute Inflammatory Polyradiculoneuropathy[Title/Abstract]) |
| 5. ((((((Multiple sclerosis[MeSH Terms]) OR (Multiple sclerosis[Title/Abstract])) OR (Sclerosis, Multiple[Title/Abstract])) OR (Sclerosis, Disseminated[Title/Abstract])) OR (Disseminated Sclerosis[Title/Abstract])) OR (MS (Multiple Sclerosis[Title/Abstract]))) OR (Multiple Sclerosis, Acute Fulminating[Title/Abstract]) |
| 6. (((((((((((((((Myasthenia gravis[MeSH Terms]) OR (Myasthenia gravis[Title/Abstract])) OR (Myasthenia Gravis, Ocular[Title/Abstract])) OR (Ocular Myasthenia Gravis[Title/Abstract])) OR (Myasthenia Gravis, Generalized[Title/Abstract])) OR (Generalized Myasthenia Gravis[Title/Abstract])) OR (Muscle-Specific Receptor Tyrosine Kinase Myasthenia Gravis[Title/Abstract])) OR (Muscle Specific Receptor Tyrosine Kinase Myasthenia Gravis[Title/Abstract])) OR (Muscle-Specific Tyrosine Kinase Antibody Positive Myasthenia Gravis[Title/Abstract])) OR (Muscle Specific Tyrosine Kinase Antibody Positive Myasthenia Gravis[Title/Abstract])) OR (MuSK MG[Title/Abstract])) OR (MuSK Myasthenia Gravis[Title/Abstract])) OR (Myasthenia Gravis, MuSK[Title/Abstract])) OR (Anti-MuSK Myasthenia Gravis[Title/Abstract])) OR (Anti MuSK Myasthenia Gravis[Title/Abstract])) OR (Myasthenia Gravis, Anti-MuSK[Title/Abstract]) |
| 7. ((((((((((((((((Addison Disease[MeSH Terms])) OR (Addison Disease[Title/Abstract])) OR (Disease, Addison[Title/Abstract])) OR (Primary Adrenal Insufficiency[Title/Abstract])) OR (Adrenal Insufficiency, Primary[Title/Abstract])) OR (Primary Hypoadrenalism[Title/Abstract])) OR (Hypoadrenalism, Primary[Title/Abstract])) OR (Addison's Disease[Title/Abstract])) OR (Addisons Disease[Title/Abstract])) OR (Primary Adrenocortical Insufficiency[Title/Abstract])) OR (Adrenocortical Insufficiencies, Primary[Title/Abstract])) OR (Adrenocortical Insufficiency, Primary[Title/Abstract])) OR (Insufficiencies, Primary Adrenocortical[Title/Abstract])) OR (Insufficiency, Primary Adrenocortical[Title/Abstract])) OR (Primary Adrenocortical Insufficiencies[Title/Abstract]) |
| 8. ((((((((((((((((((((((((((((Diabetes mellitus, type 1[MeSH Terms]) OR (Diabetes mellitus, type 1[Title/Abstract])) OR (Diabetes Mellitus, Insulin-Dependent[Title/Abstract])) OR (Diabetes Mellitus, Insulin Dependent[Title/Abstract])) OR (Insulin-Dependent Diabetes Mellitus[Title/Abstract])) OR (Diabetes Mellitus, Juvenile-Onset[Title/Abstract])) OR (Diabetes Mellitus, Juvenile Onset[Title/Abstract])) OR (Juvenile-Onset Diabetes Mellitus[Title/Abstract])) OR (IDDM[Title/Abstract])) OR (Juvenile-Onset Diabetes[Title/Abstract])) OR (Diabetes, Juvenile-Onset[Title/Abstract])) OR (Juvenile Onset Diabetes[Title/Abstract])) OR (Diabetes Mellitus, Sudden-Onset[Title/Abstract])) OR (Diabetes Mellitus, Sudden Onset[Title/Abstract])) OR (Sudden-Onset Diabetes Mellitus[Title/Abstract])) OR (Type 1 Diabetes Mellitus[Title/Abstract])) OR (Diabetes Mellitus, Insulin-Dependent, 1[Title/Abstract])) OR (Insulin-Dependent Diabetes Mellitus 1[Title/Abstract])) OR (Insulin Dependent Diabetes Mellitus 1[Title/Abstract])) OR (Type 1 Diabetes[Title/Abstract])) OR (Diabetes, Type 1[Title/Abstract])) OR (Diabetes Mellitus, Type I[Title/Abstract])) OR (Diabetes, Autoimmune[Title/Abstract])) OR (Autoimmune Diabetes[Title/Abstract])) OR (Diabetes Mellitus, Brittle[Title/Abstract])) OR (Brittle Diabetes Mellitus[Title/Abstract])) OR (Diabetes Mellitus, Ketosis-Prone[Title/Abstract])) OR (Diabetes Mellitus, Ketosis Prone[Title/Abstract])) OR (Ketosis-Prone Diabetes Mellitus[Title/Abstract]) |
| 9. ((((((((((((((Graves Disease[MeSH Terms]) OR (Graves Disease[Title/Abstract])) OR (Disease, Graves[Title/Abstract])) OR (Basedow Disease[Title/Abstract])) OR (Disease, Basedow[Title/Abstract])) OR (Graves' Disease[Title/Abstract])) OR (Disease, Graves'[Title/Abstract])) OR (Exophthalmic Goiter[Title/Abstract])) OR (Exophthalmic Goiters[Title/Abstract])) OR (Goiters, Exophthalmic[Title/Abstract])) OR (Goiter, Exophthalmic[Title/Abstract])) OR (Hyperthyroidism, Autoimmune[Title/Abstract])) OR (Basedow's Disease[Title/Abstract])) OR (Basedows Disease[Title/Abstract])) OR (Disease, Basedow's[Title/Abstract]) |
| 10. (((((((((((((((((((((((Hashimoto Disease[MeSH Terms]) OR (Hashimoto Disease[Title/Abstract])) OR (Disease, Hashimoto[Title/Abstract])) OR (Hashimoto Struma[Title/Abstract])) OR (Hashimoto Thyroiditis[Title/Abstract])) OR (Hashimoto Thyroiditides[Title/Abstract])) OR (Thyroiditides, Hashimoto[Title/Abstract])) OR (Thyroiditis, Hashimoto[Title/Abstract])) OR (Hashimoto's Syndrome[Title/Abstract])) OR (Hashimoto Syndrome[Title/Abstract])) OR (Hashimoto's Syndromes[Title/Abstract])) OR (Hashimotos Syndrome[Title/Abstract])) OR (Syndrome, Hashimoto's[Title/Abstract])) OR (Syndromes, Hashimoto's[Title/Abstract])) OR (Hashimoto's Struma[Title/Abstract])) OR (Chronic Lymphocytic Thyroiditis[Title/Abstract])) OR (Chronic Lymphocytic Thyroiditides[Title/Abstract])) OR (Lymphocytic Thyroiditides, Chronic[Title/Abstract])) OR (Lymphocytic Thyroiditis, Chronic[Title/Abstract])) OR (Thyroiditides, Chronic Lymphocytic[Title/Abstract])) OR (Thyroiditis, Chronic Lymphocytic[Title/Abstract])) OR (Hashimoto's Disease[Title/Abstract])) OR (Disease, Hashimoto's[Title/Abstract])) OR (Hashimotos Disease[Title/Abstract]) |
| 11. ((((((((((Hepatitis, Autoimmune[MeSH Terms]) OR (Hepatitis, Autoimmune[Title/Abstract])) OR (Autoimmune Hepatitides[Title/Abstract])) OR (Hepatitides, Autoimmune[Title/Abstract])) OR (Autoimmune Chronic Hepatitis[Title/Abstract])) OR (Autoimmune Chronic Hepatitides[Title/Abstract])) OR (Chronic Hepatitides, Autoimmune[Title/Abstract])) OR (Chronic Hepatitis, Autoimmune[Title/Abstract])) OR (Hepatitides, Autoimmune Chronic[Title/Abstract])) OR (Hepatitis, Autoimmune Chronic[Title/Abstract])) OR (Autoimmune Hepatitis[Title/Abstract]) |
| 12. (((Inflammatory Bowel Diseases[MeSH Terms]) OR (Inflammatory Bowel Diseases[Title/Abstract])) OR (Inflammatory Bowel Disease[Title/Abstract])) OR (Bowel Diseases, Inflammatory[Title/Abstract]) |
| 13. (((((((((((((((((Crohn Disease[MeSH Terms]) OR (Crohn Disease[Title/Abstract])) OR (Crohn's Enteritis[Title/Abstract])) OR (Regional Enteritis[Title/Abstract])) OR (Crohn's Disease[Title/Abstract])) OR (Crohns Disease[Title/Abstract])) OR (Inflammatory Bowel Disease 1[Title/Abstract])) OR (Enteritis, Granulomatous[Title/Abstract])) OR (Granulomatous Enteritis[Title/Abstract])) OR (Enteritis, Regional[Title/Abstract])) OR (Ileocolitis[Title/Abstract])) OR (Colitis, Granulomatous[Title/Abstract])) OR (Granulomatous Colitis[Title/Abstract])) OR (Ileitis, Terminal[Title/Abstract])) OR (Terminal Ileitis[Title/Abstract])) OR (Ileitis, Regional[Title/Abstract])) OR (Regional Ileitides[Title/Abstract])) OR (Regional Ileitis[Title/Abstract]) |
| 14. (((((Colitis, Ulcerative[MeSH Terms]) OR (Colitis, Ulcerative[Title/Abstract])) OR (Idiopathic Proctocolitis[Title/Abstract])) OR (Ulcerative Colitis[Title/Abstract])) OR (Colitis Gravis[Title/Abstract])) OR (Inflammatory Bowel Disease, Ulcerative Colitis Type[Title/Abstract]) |
| 15. ((((((((((((((((Coeliac disease[MeSH Terms]) OR (Coeliac disease[Title/Abstract])) OR (Disease, Celiac[Title/Abstract])) OR (Gluten Enteropathy[Title/Abstract])) OR (Enteropathies, Gluten[Title/Abstract])) OR (Enteropathy, Gluten[Title/Abstract])) OR (Gluten Enteropathies[Title/Abstract])) OR (Gluten-Sensitive Enteropathy[Title/Abstract])) OR (Enteropathies, Gluten-Sensitive[Title/Abstract])) OR (Enteropathy, Gluten-Sensitive[Title/Abstract])) OR (Gluten Sensitive Enteropathy[Title/Abstract])) OR (Gluten-Sensitive Enteropathies[Title/Abstract])) OR (Sprue, Celiac[Title/Abstract])) OR (Sprue, Nontropical[Title/Abstract])) OR (Nontropical Sprue[Title/Abstract])) OR (Celiac Sprue[Title/Abstract])) OR (Sprue[Title/Abstract]) |
| 16. ((((((((Anemia, Pernicious[MeSH Terms]) OR (Anemia, Pernicious[Title/Abstract])) OR (Pernicious Anemia[Title/Abstract])) OR (Anemia, Addison's[Title/Abstract])) OR (Anemia, Addison[Title/Abstract])) OR (Anemia, Addisons[Title/Abstract])) OR (Addison's Anemia[Title/Abstract])) OR (Addison Anemia[Title/Abstract])) OR (Addisons Anemia[Title/Abstract]) |
| 17. (((((((((((((((((((((((Liver Cirrhosis, Biliary[MeSH Terms]) OR (Liver Cirrhosis, Biliary[Title/Abstract])) OR (Liver Cirrhoses, Biliary[Title/Abstract])) OR (Biliary Cirrhosis[Title/Abstract])) OR (Cirrhosis, Biliary[Title/Abstract])) OR (Biliary Cirrhosis, Primary, 1[Title/Abstract])) OR (Secondary Biliary Cholangitis[Title/Abstract])) OR (Biliary Cholangitis, Secondary[Title/Abstract])) OR (Cholangitis, Secondary Biliary[Title/Abstract])) OR (Secondary Biliary Cholangitides[Title/Abstract])) OR (Liver Cirrhosis, Obstructive[Title/Abstract])) OR (Obstructive Liver Cirrhosis[Title/Abstract])) OR (Secondary Biliary Cirrhosis[Title/Abstract])) OR (Biliary Cirrhosis, Secondary[Title/Abstract])) OR (Cirrhosis, Secondary Biliary[Title/Abstract])) OR (Primary Biliary Cholangitis[Title/Abstract])) OR (Biliary Cholangitides, Primary[Title/Abstract])) OR (Biliary Cholangitis, Primary[Title/Abstract])) OR (Cholangitides, Primary Biliary[Title/Abstract])) OR (Cholangitis, Primary Biliary[Title/Abstract])) OR (Primary Biliary Cholangitides[Title/Abstract])) OR (Biliary Cirrhosis, Primary[Title/Abstract])) OR (Cholangitis, Chronic Nonsuppurative Destructive[Title/Abstract])) OR (Primary Biliary Cirrhosis[Title/Abstract]) |
| 18. ((alopecia areata[MeSH Terms]) OR (alopecia areata[Title/Abstract])) OR (Alopecia Circumscripta[Title/Abstract]) |
| 19. ((((((((((((((Cholangitis, Sclerosing[MeSH Terms]) OR (Cholangitis, Sclerosing[Title/Abstract])) OR (Cholangitides, Sclerosing[Title/Abstract])) OR (Sclerosing Cholangitides[Title/Abstract])) OR (Sclerosing Cholangitis[Title/Abstract])) OR (Cholangiitis, Sclerosing[Title/Abstract])) OR (Cholangiitides, Sclerosing[Title/Abstract])) OR (Sclerosing Cholangiitides[Title/Abstract])) OR (Sclerosing Cholangiitis[Title/Abstract])) OR (Primary Sclerosing Cholangitis[Title/Abstract])) OR (Cholangitides, Primary Sclerosing[Title/Abstract])) OR (Primary Sclerosing Cholangitides[Title/Abstract])) OR (Sclerosing Cholangitides, Primary[Title/Abstract])) OR (Sclerosing Cholangitis, Primary[Title/Abstract])) OR (Cholangitis, Primary Sclerosing[Title/Abstract]) |
| 20. (((((((((((((((Antiphospholipid syndrome[MeSH Terms]) OR (Antiphospholipid syndrome[Title/Abstract])) OR (Syndrome, Antiphospholipid[Title/Abstract])) OR (Hughes Syndrome[Title/Abstract])) OR (Syndrome, Hughes[Title/Abstract])) OR (Antiphospholipid Antibody Syndrome[Title/Abstract])) OR (Antibody Syndrome, Antiphospholipid[Title/Abstract])) OR (Antiphospholipid Antibody Syndromes[Title/Abstract])) OR (Syndrome, Antiphospholipid Antibody[Title/Abstract])) OR (Anti-Phospholipid Antibody Syndrome[Title/Abstract])) OR (Anti Phospholipid Antibody Syndrome[Title/Abstract])) OR (Antibody Syndrome, Anti-Phospholipid[Title/Abstract])) OR (Syndrome, Anti-Phospholipid Antibody[Title/Abstract])) OR (Anti-Phospholipid Syndrome[Title/Abstract])) OR (Anti Phospholipid Syndrome[Title/Abstract])) OR (Syndrome, Anti-Phospholipid[Title/Abstract]) |
| 21. (((((((((((((((((((Anemia, Hemolytic, Autoimmune[MeSH Terms]) OR (Anemia, Hemolytic, Autoimmune[Title/Abstract])) OR (Hemolytic Anemia, Autoimmune[Title/Abstract])) OR (Autoimmune Hemolytic Anemia[Title/Abstract])) OR (Anemia, Autoimmune Hemolytic[Title/Abstract])) OR (Autoimmune Hemolytic Anemias[Title/Abstract])) OR (Autoimmune Haemolytic Anaemia[Title/Abstract])) OR (Anaemia, Autoimmune Haemolytic[Title/Abstract])) OR (Autoimmune Haemolytic Anaemias[Title/Abstract])) OR (Haemolytic Anaemia, Autoimmune[Title/Abstract])) OR (Cold Agglutinin Disease[Title/Abstract])) OR (Agglutinin Disease, Cold[Title/Abstract])) OR (Cold Agglutinin Diseases[Title/Abstract])) OR (Cold Antibody Disease[Title/Abstract])) OR (Cold Antibody Diseases[Title/Abstract])) OR (Cold Antibody Hemolytic Anemia[Title/Abstract])) OR (Anemia, Hemolytic, Cold Antibody[Title/Abstract])) OR (Idiopathic Autoimmune Hemolytic Anemia[Title/Abstract])) OR (Acquired Autoimmune Hemolytic Anemia[Title/Abstract])) OR (Anemia, Hemolytic, Idiopathic Acquired[Title/Abstract]) |
| 22. (((((((((((((((((((((((((((((((Purpura, Thrombocytopenic, Idiopathic[MeSH Terms]) OR (Purpura, Thrombocytopenic, Idiopathic[Title/Abstract])) OR (Idiopathic Thrombocytopenic Purpura[Title/Abstract])) OR (Idiopathic Thrombocytopenic Purpuras[Title/Abstract])) OR (Purpura, Idiopathic Thrombocytopenic[Title/Abstract])) OR (Purpuras, Idiopathic Thrombocytopenic[Title/Abstract])) OR (Thrombocytopenic Purpura, Idiopathic[Title/Abstract])) OR (Thrombocytopenic Purpuras, Idiopathic[Title/Abstract])) OR (Immune Thrombocytopenic Purpura[Title/Abstract])) OR (Immune Thrombocytopenic Purpuras[Title/Abstract])) OR (Purpura, Immune Thrombocytopenic[Title/Abstract])) OR (Purpuras, Immune Thrombocytopenic[Title/Abstract])) OR (Thrombocytopenic Purpura, Immune[Title/Abstract])) OR (Thrombocytopenic Purpuras, Immune[Title/Abstract])) OR (Immune Thrombocytopenia[Title/Abstract])) OR (Immune Thrombocytopenias[Title/Abstract])) OR (Thrombocytopenia, Immune[Title/Abstract])) OR (Thrombocytopenias, Immune[Title/Abstract])) OR (Thrombocytopenic Purpura, Autoimmune[Title/Abstract])) OR (Werlhof Disease[Title/Abstract])) OR (Disease, Werlhof[Title/Abstract])) OR (Werlhof's Disease[Title/Abstract])) OR (Disease, Werlhof's[Title/Abstract])) OR (Autoimmune Thrombocytopenia[Title/Abstract])) OR (Autoimmune Thrombocytopenias[Title/Abstract])) OR (Thrombocytopenia, Autoimmune[Title/Abstract])) OR (Thrombocytopenias, Autoimmune[Title/Abstract])) OR (Autoimmune Thrombocytopenic Purpura[Title/Abstract])) OR (Autoimmune Thrombocytopenic Purpuras[Title/Abstract])) OR (Purpura, Autoimmune Thrombocytopenic[Title/Abstract])) OR (Purpuras, Autoimmune Thrombocytopenic[Title/Abstract])) OR (Purpura, Thrombocytopenic, Autoimmune[Title/Abstract]) |
| 23. ((((((((((((Dermatomyositis[MeSH Terms]) OR (Dermatomyositis[Title/Abstract])) OR (Dermatopolymyositis[Title/Abstract])) OR (Polymyositis-Dermatomyositis[Title/Abstract])) OR (Polymyositis Dermatomyositis[Title/Abstract])) OR (Dermatomyositis, Adult Type[Title/Abstract])) OR (Adult Type Dermatomyositis[Title/Abstract])) OR (Dermatomyositis, Childhood Type[Title/Abstract])) OR (Childhood Type Dermatomyositis[Title/Abstract])) OR (Juvenile Dermatomyositis[Title/Abstract])) OR (Dermatomyositis, Juvenile[Title/Abstract])) OR (Juvenile Myositis[Title/Abstract])) OR (Myositis, Juvenile[Title/Abstract]) |
| 24. ((Arthritis, Rheumatoid[MeSH Terms]) OR (Arthritis, Rheumatoid[Title/Abstract])) OR (Rheumatoid Arthritis[Title/Abstract]) |
| 25. ((((((((Polyarteritis nodosa[MeSH Terms]) OR (Polyarteritis nodosa[Title/Abstract])) OR (Periarteritis Nodosa[Title/Abstract])) OR (Necrotizing Arteritis[Title/Abstract])) OR (Arteritides, Necrotizing[Title/Abstract])) OR (Arteritis, Necrotizing[Title/Abstract])) OR (Necrotizing Arteritides[Title/Abstract])) OR (Essential Polyarteritis[Title/Abstract])) OR (Polyarteritis, Essential[Title/Abstract]) |
| 26. (((((((((((((((((((((((((((((((((Giant Cell Arteritis[MeSH Terms]) OR (Giant Cell Arteritis[Title/Abstract])) OR (Arteritides, Giant Cell[Title/Abstract])) OR (Arteritis, Giant Cell[Title/Abstract])) OR (Giant Cell Arteritides[Title/Abstract])) OR (Arteritis, Giant Cell, Horton's[Title/Abstract])) OR (Horton's Giant Cell Arteritis[Title/Abstract])) OR (Horton Disease[Title/Abstract])) OR (Horton Giant Cell Arteritis[Title/Abstract])) OR (Horton's Disease[Title/Abstract])) OR (Hortons Disease[Title/Abstract])) OR (Arteritis, Giant Cell, Horton[Title/Abstract])) OR (Giant Cell Arteritis, Horton[Title/Abstract])) OR (Temporal Arteritis[Title/Abstract])) OR (Arteritides, Temporal[Title/Abstract])) OR (Temporal Arteritides[Title/Abstract])) OR (Arteritis, Temporal[Title/Abstract])) OR (Juvenile Temporal Arteritis[Title/Abstract])) OR (Arteritides, Juvenile Temporal[Title/Abstract])) OR (Arteritis, Juvenile Temporal[Title/Abstract])) OR (Juvenile Temporal Arteritides[Title/Abstract])) OR (Temporal Arteritides, Juvenile[Title/Abstract])) OR (Temporal Arteritis, Juvenile[Title/Abstract])) OR (Aortic Arteritis, Giant Cell[Title/Abstract])) OR (Giant Cell Aortitis, Horton's[Title/Abstract])) OR (Giant Cell Aortitis[Title/Abstract])) OR (Aortitis, Giant Cell[Title/Abstract])) OR (Aortitides, Giant Cell[Title/Abstract])) OR (Giant Cell Aortitides[Title/Abstract])) OR (Giant Cell Aortic Arteritis[Title/Abstract])) OR (Cranial Arteritis[Title/Abstract])) OR (Arteritides, Cranial[Title/Abstract])) OR (Arteritis, Cranial[Title/Abstract])) OR (Cranial Arteritides[Title/Abstract]) |
| 27. ((((Pemphigoid, Bullous[MeSH Terms]) OR (Pemphigoid, Bullous[Title/Abstract])) OR (Bullous Pemphigoid[Title/Abstract])) OR (Pemphigoid[Title/Abstract])) OR (Pemphigoids[Title/Abstract]) |
| 28. ((((Lupus Erythematosus, Discoid[MeSH Terms]) OR (Lupus Erythematosus, Discoid[Title/Abstract])) OR (Discoid Lupus Erythematosus[Title/Abstract])) OR (Lupus Erythematosus, Chronic Cutaneous[Title/Abstract])) OR (Lupus Erythematosus, Cutaneous, Chronic[Title/Abstract]) |
| 29. (Vitiligo[MeSH Terms]) OR (Vitiligo[Title/Abstract]) |
| 30. ((((((((((((((Behcet Syndrome[MeSH Terms]) OR (Behcet Syndrome[Title/Abstract])) OR (Behcet's Syndrome[Title/Abstract])) OR (Triple-Symptom Complex[Title/Abstract])) OR (Triple Symptom Complex[Title/Abstract])) OR (Symptom Complex, Triple[Title/Abstract])) OR (Behçet Disease[Title/Abstract])) OR (Behçet Diseases[Title/Abstract])) OR (Adamantiades-Behcet Disease[Title/Abstract])) OR (Adamantiades Behcet Disease[Title/Abstract])) OR (Adamantiades-Behcet Diseases[Title/Abstract])) OR (Behcet Triple Symptom Complex[Title/Abstract])) OR (Old Silk Route Disease[Title/Abstract])) OR (Behcet's Disease[Title/Abstract])) OR (Behcet Disease[Title/Abstract]) |
| 31. ((((((((((((((Scleroderma[MeSH Terms]) OR (Scleroderma[Title/Abstract])) OR (Localized Scleroderma[Title/Abstract])) OR (Sclerodermas, Localized[Title/Abstract])) OR (Scleroderma, Circumscribed[Title/Abstract])) OR (Circumscribed Scleroderma[Title/Abstract])) OR (Dermatosclerosis[Title/Abstract])) OR (Morphea[Title/Abstract])) OR (Morpheas[Title/Abstract])) OR (Scleroderma, Linear[Title/Abstract])) OR (Linear Scleroderma[Title/Abstract])) OR (Frontal Linear Scleroderma en Coup de Sabre[Title/Abstract])) OR (Systemic Sclerosis[Title/Abstract])) OR (Sclerosis, Systemic[Title/Abstract])) OR (Systemic Scleroderma[Title/Abstract]) |
| 32. ((((((Sjogren's Syndrome[MeSH Terms]) OR (Sjogren's Syndrome[Title/Abstract])) OR (Sjogrens Syndrome[Title/Abstract])) OR (Syndrome, Sjogren's[Title/Abstract])) OR (Sjogren Syndrome[Title/Abstract])) OR (Sicca Syndrome[Title/Abstract])) OR (Syndrome, Sicca[Title/Abstract]) |
| 33. ((((((Lupus Erythematosus, Systemic[MeSH Terms]) OR (Lupus Erythematosus, Systemic[Title/Abstract])) OR (Systemic Lupus Erythematosus[Title/Abstract])) OR (Lupus Erythematosus Disseminatus[Title/Abstract])) OR (Libman-Sacks Disease[Title/Abstract])) OR (Disease, Libman-Sacks[Title/Abstract])) OR (Libman Sacks Disease[Title/Abstract]) |
| 34. ((((((Dermatitis Herpetiformis[MeSH Terms]) OR (Dermatitis Herpetiformis[Title/Abstract])) OR (Duhring's Disease[Title/Abstract])) OR (Disease, Duhring's[Title/Abstract])) OR (Duhrings Disease[Title/Abstract])) OR (Duhring Disease[Title/Abstract])) OR (Disease, Duhring[Title/Abstract]) |
| 35. (((((((((((((((((((Narcolepsy[MeSH Terms]) OR (Narcolepsy[Title/Abstract])) OR (Paroxysmal Sleep[Title/Abstract])) OR (Sleep, Paroxysmal[Title/Abstract])) OR (Narcoleptic Syndrome[Title/Abstract])) OR (Narcoleptic Syndromes[Title/Abstract])) OR (Syndrome, Narcoleptic[Title/Abstract])) OR (Syndromes, Narcoleptic[Title/Abstract])) OR (Gelineau Syndrome[Title/Abstract])) OR (Syndrome, Gelineau[Title/Abstract])) OR (Gelineau's Syndrome[Title/Abstract])) OR (Gelineau's Syndromes[Title/Abstract])) OR (Syndrome, Gelineau's[Title/Abstract])) OR (Syndromes, Gelineau's[Title/Abstract])) OR (Narcolepsy-Cataplexy Syndrome[Title/Abstract])) OR (Narcolepsy Cataplexy Syndrome[Title/Abstract])) OR (Narcolepsy-Cataplexy Syndromes[Title/Abstract])) OR (Syndrome, Narcolepsy-Cataplexy[Title/Abstract])) OR (Syndromes, Narcolepsy-Cataplexy[Title/Abstract]) |
| 36. ((((((((((((((((((((((((((((((((Rheumatic Fever[MeSH Terms]) OR (Fever, Rheumatic[Title/Abstract])) OR (Fevers, Rheumatic[Title/Abstract])) OR (Rheumatic Fevers[Title/Abstract])) OR (Arthritis, Acute Rheumatic[Title/Abstract])) OR (Acute Rheumatic Arthritides[Title/Abstract])) OR (Acute Rheumatic Arthritis[Title/Abstract])) OR (Arthritides, Acute Rheumatic[Title/Abstract])) OR (Rheumatic Arthritides, Acute[Title/Abstract])) OR (Rheumatic Arthritis, Acute[Title/Abstract])) OR (Arthritis, Rheumatic, Acute[Title/Abstract])) OR (Rheumatism, Articular, Acute[Title/Abstract])) OR (Polyarthritis Rheumatica[Title/Abstract])) OR (Rheumatic Arthritis[Title/Abstract])) OR (Arthritides, Rheumatic[Title/Abstract])) OR (Arthritis, Rheumatic[Title/Abstract])) OR (Rheumatic Arthritides[Title/Abstract])) OR (Rheumatism, Acute Articular[Title/Abstract])) OR (Acute Articular Rheumatism[Title/Abstract])) OR (Acute Articular Rheumatisms[Title/Abstract])) OR (Articular Rheumatism, Acute[Title/Abstract])) OR (Articular Rheumatisms, Acute[Title/Abstract])) OR (Rheumatisms, Acute Articular[Title/Abstract])) OR (Acute Rheumatic Fever[Title/Abstract])) OR (Acute Rheumatic Fevers[Title/Abstract])) OR (Fever, Acute Rheumatic[Title/Abstract])) OR (Fevers, Acute Rheumatic[Title/Abstract])) OR (Rheumatic Fever, Acute[Title/Abstract])) OR (Rheumatic Fevers, Acute[Title/Abstract])) OR (Inflammatory Rheumatism[Title/Abstract])) OR (Inflammatory Rheumatisms[Title/Abstract])) OR (Rheumatism, Inflammatory[Title/Abstract])) OR (Rheumatisms, Inflammatory[Title/Abstract]) |
| 37. 2 OR 3 OR 4 OR 5 OR 6 OR 7 OR 8 OR 9 OR 10 OR 11 OR 12 OR 13 OR 14 OR 15 OR 16 OR 17 OR 18 OR 19 OR 20 OR 21 OR 22 OR 23 OR 24 OR 25 OR 26 OR 27 OR 28 OR 29 OR 30 OR 31 OR 32 OR 33 OR 34 OR 35 OR 36 |
| 38. 1 and 37 |

**Table 2.** Quality assessment.

Study quality assessment using Newcastle-Ottawa scale for cohort studies (part 1).

| Study | Selection | | | | Comparability | Outcome | | | Total |
| --- | --- | --- | --- | --- | --- | --- | --- | --- | --- |
|  | Representativeness  of exposed cohort | Selection of  nonexposed  cohort | Ascertainment of exposure | Outcome not  present at  baseline |  | Assessment of outcome | Sufficient  follow-up  duration | Adequate  follow-up |  |
| Ludvigsson et al. (2007) | 1 | 1 | 1 | 1 | 1 | 1 | 1 | 1 | 8 |
| Li et al. (2012) | 1 | 0 | 1 | 1 | 2 | 1 | 1 | 0 | 7 |
| Brick et al. (2014) | 1 | 1 | 1 | 1 | 1 | 1 | 1 | 1 | 8 |
| Nielsen et al. (2014) | 1 | 1 | 1 | 0 | 1 | 1 | 1 | 0 | 6 |
| Hsu et al. (2015) | 1 | 1 | 1 | 1 | 1 | 1 | 0 | 1 | 7 |
| Liu et al. (2015) | 1 | 1 | 1 | 1 | 2 | 1 | 1 | 1 | 9 |
| Thormann et al. (2016) | 1 | 1 | 1 | 1 | 1 | 1 | 1 | 0 | 7 |
| Lin et al. (2016) | 1 | 1 | 1 | 1 | 2 | 1 | 1 | 1 | 9 |
| Sung et al. (2016) | 1 | 1 | 1 | 1 | 2 | 1 | 1 | 1 | 9 |
| Kibsgaard et al. (2017) | 1 | 1 | 1 | 1 | 1 | 1 | 0 | 1 | 7 |
| Chang et al. (2018) | 1 | 1 | 1 | 1 | 2 | 1 | 1 | 1 | 9 |
| Peter et al. (2018) | 1 | 1 | 1 | 1 | 2 | 1 | 1 | 1 | 9 |
| Villumsen et al. (2018) | 1 | 1 | 1 | 1 | 2 | 1 | 1 | 1 | 9 |
| Chen et al. (2019) | 1 | 1 | 1 | 1 | 1 | 1 | 1 | 1 | 8 |
| Ju et al. (2019) | 1 | 1 | 1 | 1 | 2 | 1 | 1 | 1 | 9 |
| Park et al. (2019) a | 1 | 1 | 1 | 1 | 2 | 1 | 1 | 1 | 9 |
| Weimers et al. (2019) | 1 | 1 | 1 | 1 | 2 | 1 | 1 | 1 | 9 |
| Hsu et al. (2020) | 1 | 1 | 1 | 1 | 2 | 1 | 1 | 1 | 9 |
| Coates et al. (2021) | 1 | 1 | 1 | 1 | 2 | 1 | 1 | 1 | 9 |
| Cho et al. (2022) | 1 | 0 | 1 | 1 | 2 | 1 | 1 | 0 | 7 |
| Kim et al. (2022) | 1 | 1 | 1 | 1 | 2 | 1 | 1 | 1 | 9 |
| Kwon et al (2022) | 1 | 1 | 1 | 1 | 2 | 1 | 1 | 1 | 9 |

Study quality assessment using Newcastle-Ottawa scale for case-control studies (part 2).

| Study | Selection | | | | Comparability | Outcome | | | Total |
| --- | --- | --- | --- | --- | --- | --- | --- | --- | --- |
|  | Case definition | Representativeness of the cases | Selection of controls | Definition of controls |  | Ascertainment of exposure | Same method of ascertainment | Non-response rate |  |
| Kronzer et al. (2009) | 1 | 1 | 1 | 1 | 2 | 1 | 1 | 0 | 8 |
| Rugbjerg et al. (2009) | 1 | 1 | 1 | 1 | 1 | 1 | 1 | 0 | 7 |
| Taghipour et al. (2010) | 1 | 1 | 1 | 0 | 2 | 1 | 1 | 1 | 8 |
| Bastuji-Garin et al. (2011) | 1 | 1 | 1 | 0 | 1 | 1 | 1 | 1 | 7 |
| Chen et al. (2011) | 1 | 1 | 1 | 0 | 2 | 1 | 1 | 1 | 8 |
| Langan et al. (2011) | 1 | 1 | 1 | 1 | 2 | 1 | 1 | 1 | 9 |
| Brick et al. (2014) | 1 | 1 | 1 | 1 | 1 | 1 | 1 | 1 | 8 |
| Teixeira et al. (2014) | 1 | 1 | 1 | 0 | 1 | 1 | 1 | 0 | 6 |
| Bählera et al. (2017) | 1 | 0 | 0 | 1 | 2 | 1 | 1 | 1 | 7 |
| Daneshpazhooh et al. (2017) | 1 | 1 | 1 | 0 | 1 | 1 | 1 | 1 | 7 |
| Khosravani et al. (2017) | 1 | 1 | 1 | 0 | 1 | 1 | 1 | 0 | 6 |
| Sim et al. (2017) | 1 | 1 | 1 | 0 | 1 | 1 | 1 | 1 | 7 |
| Wu et al. (2017) | 1 | 1 | 1 | 1 | 1 | 1 | 1 | 0 | 7 |
| Yu Phuan et al. (2017) | 1 | 1 | 1 | 1 | 2 | 1 | 1 | 1 | 9 |
| Camacho-soto et al. (2018) | 1 | 0 | 0 | 1 | 2 | 1 | 1 | 1 | 7 |
| Jeon et al. (2018) | 1 | 1 | 1 | 0 | 0 | 1 | 1 | 1 | 6 |
| Kridin et al. (2018) | 1 | 1 | 1 | 0 | 2 | 1 | 1 | 1 | 8 |
| Papakonstantinou et al. (2019) | 1 | 1 | 1 | 0 | 1 | 1 | 1 | 1 | 7 |
| Bacelis et al. (2021) | 1 | 1 | 1 | 1 | 1 | 1 | 1 | 1 | 8 |
| Kridin et al. (2021) | 1 | 1 | 1 | 0 | 1 | 1 | 1 | 1 | 7 |
| Sayar et al. (2021) | 1 | 1 | 1 | 0 | 1 | 1 | 1 | 1 | 7 |

**Table 3.** Correlation of Parkinson's disease with autoimmune disease in the excluded literature.

| Study | Measures reported | Risk estimates (in original reports) | Risk factors adjusted | Risk estimates (calculated / with correction) | Study Quality |
| --- | --- | --- | --- | --- | --- |
| Rugbjerg et al. (2009) | OR | MG: 0.83 (0.19-3.72)  PM: 1.43 (0.30-6.88)  Scleroderma: 4.00 (1.07-14.89)  Addison disease: 0.71 (0.16-3.14)  AIHA: 1.43 (0.30-6.88)  PA: 1.18 (0.73-1.90)  PBC: 1.43 (0.30-6.88) | NA | NA | 7/9 |
| Li et al. (2012) | SIR | NA | Age, period, socioeconomic status,region of residence, hospitalization of COPD, and alcoholism and alcohol-related liver disease | T1D: 3.03 (0.29-11.14)  BD: 1.33 (0.63-2.45)  MG: 1.38 (0.55-2.86)  PM: 0.87 (0.08-3.18)  Scleroderma: 0.65 (0.06-2.38)  Addison disease: 0.45 (0.00-2.59)  AIHA: 1.03 (0.10-3.79)  PA: 1.49 (1.13-1.94)  PBC: 2.90 (0.75-7.49) | 7/9 |
| Nielsen et al. (2014) | SIR | MS: 0.98 (0.67-1.44) | NA | NA | 6/9 |
| Hsu et al. (2015) | RR | IBD: 1.11 (0.77-1.61) | NA | NA | 7/9 |
| Klimek et al. (2015) | RR | T1D: 2.30 (1.90-2.70) | NA | NA | 5/9 |
| Chang et al. (2018) | HR | NA | Age group, sex, and comorbidities | SLE: 1.21 (0.91-1.61) | 9/9 |
| Ju et al. (2019) | HR | SS: 1.40 (1.32-1.48) | Age, sex, and comorbidities | SS: 1.23 (1.16-1.30) | 9/9 |
| Park et al. (2019) b | HR | BD: 2.96 (2.00-4.37) | Age, sex, and comorbidities | BD: 2.47 (1.65-3.68) | 9/9 |
| Hsu et al. (2020) | HR | SS: 1.31 (1.15-1.50) | Age group, gender, and comorbidities | SS: 1.23 (1.07-1.42) | 9/9 |
| Noh et al. (2020) | HR | IBD: 0.60 (0.37-0.96) | Sex, age, alcohol and comorbidities | IBD: 0.56 (0.34-0.92) | 9/9 |

**Table 4.** Results of Begg’s test and Egger’s test.

| Types | *p* value in Begg’s test | *p* value in Egger’s test |
| --- | --- | --- |
| All autoimmune diseases | 0.384 | 0.403 |
| Study type | | |
| Case-control study | 0.650 | 0.205 |
| Cross-sectional study | 0.734 | 0.655 |
| Cohort study | 0.130 | 0.171 |
| Gender | | |
| Male | 0.360 | 0.218 |
| Female | 0.760 | 0.314 |
| Age | | |
| <65 | 0.764 | 0.481 |
| ≥65 | 0.548 | 0.885 |
| Race | | |
| Europ | 0.194 | 0.383 |
| Asia | 0.434 | 0.258 |
| Study design | | |
| Prospective | 0.707 | 0.739 |
| Retrospective | 0.412 | 0.071 |
| Types of autoimmune diseases | | |
| BP | 0.174 | 0.956 |
| IBD | 0.917 | 0.753 |
| CD | 0.348 | 0.332 |
| UC | 0.602 | 0.518 |
| SS | 0.462 | 0.772 |
| SLE | 1.000 | 0.592 |
| MS | 0.734 | 0.888 |
| RA | 0.851 | 0.520 |
| GD | 1.000 | 0.967 |
| CLD | 1.000 | 0.488 |

**Table 5.** Sensitivity analysis, the impact of individual studies on the overall pooled result was assessed by excluding one study at a time.

| Types | OR (95% CI) | Exclude one study at a time |
| --- | --- | --- |
| All autoimmune diseases | 1.55 (1.33-1.81) | 1.55 (1.28 to 1.87) |
| BP | 2.67 (2.15-3.31) | 2.67 (2.04 to 3.51) |
| IBD | 1.30 (1.18-1.45) | 1.30 (1.15 to 1.50) |
| CD | 1.30 (1.20-1.42) | 1.30 (1.11 to 1.45) |
| UC | 1.31 (1.14-1.50) | 1.31 (1.10 to 1.56) |
| SS | 1.61 (1.24-2.09) | 1.61 (0.91 to 3.14) |
| SLE | 0.82 (0.66-1.03) | 0.82 (0.55 to 1.63) |
| MS | 2.02 (0.87-4.70) | 2.02 (0.49 to 7.92) |
| RA | 0.79 (0.61-1.03) | 0.79 (0.55 to 1.11) |
| GD | 1.45 (1.24-1.70) | 1.45 (1.18 to 1.85) |
| CLD | 1.16 (0.79-1.69) | 1.16 (0.47 to 2.26) |

**Figure 1.** The effects of the individual studies on the pooled effect size of PD and AIDs risk.

**
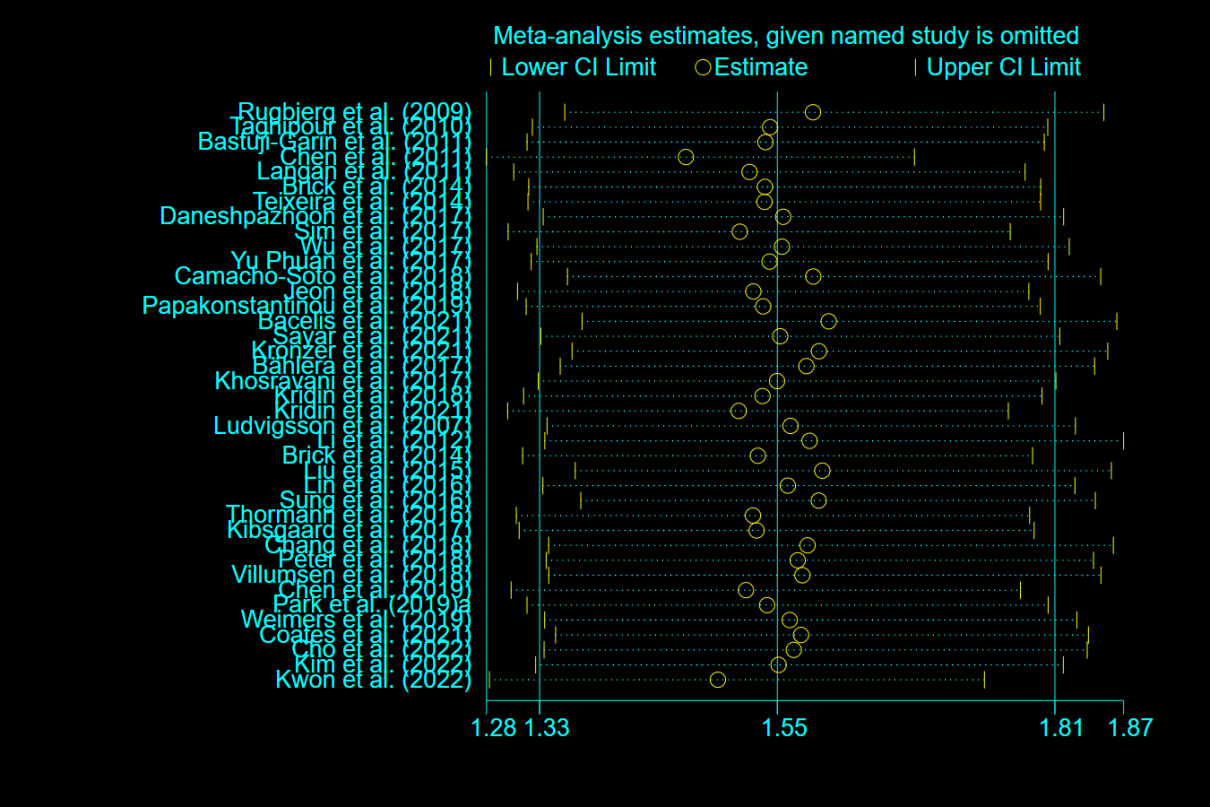
**

**A:** PD and all AIDs

**
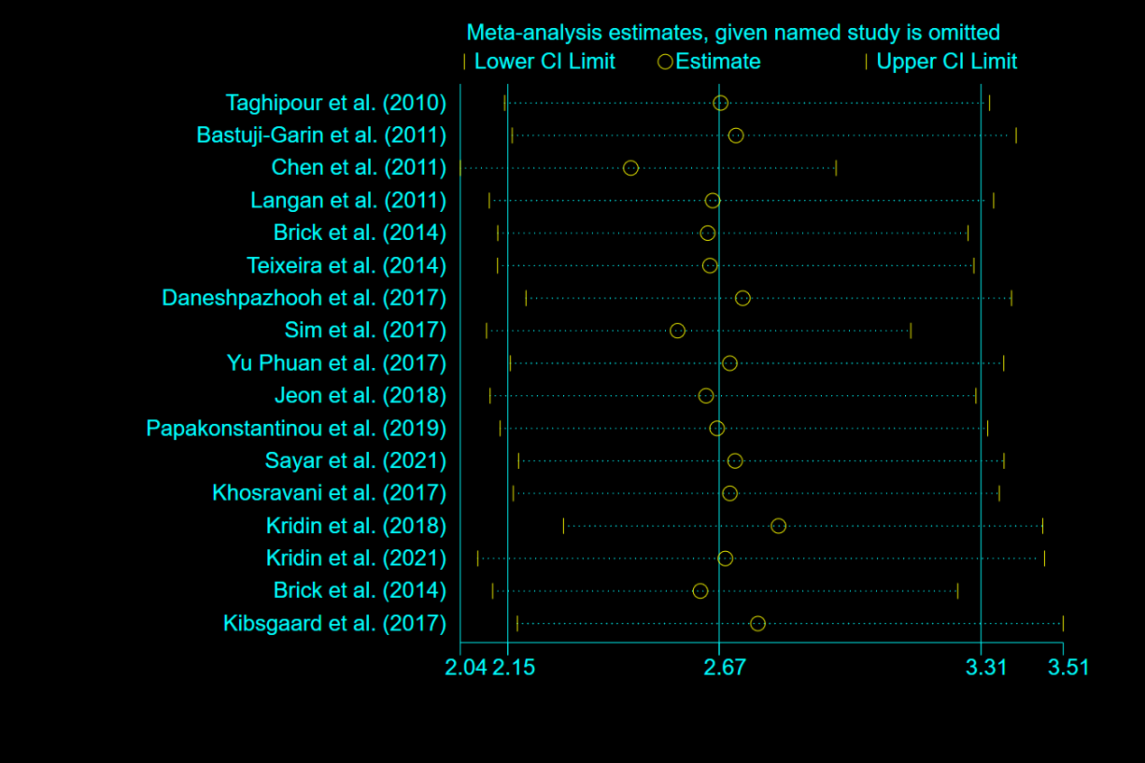
**

**B:** PD and BP

**
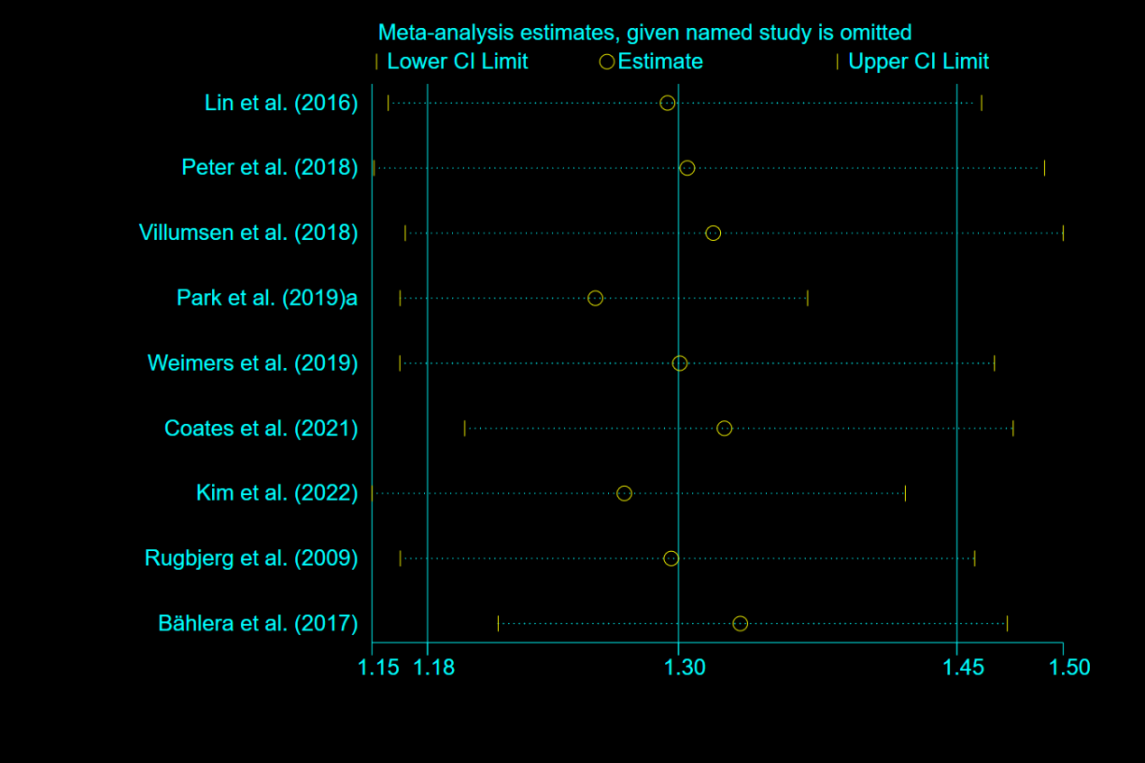
**

**C:** PD and IBD

**
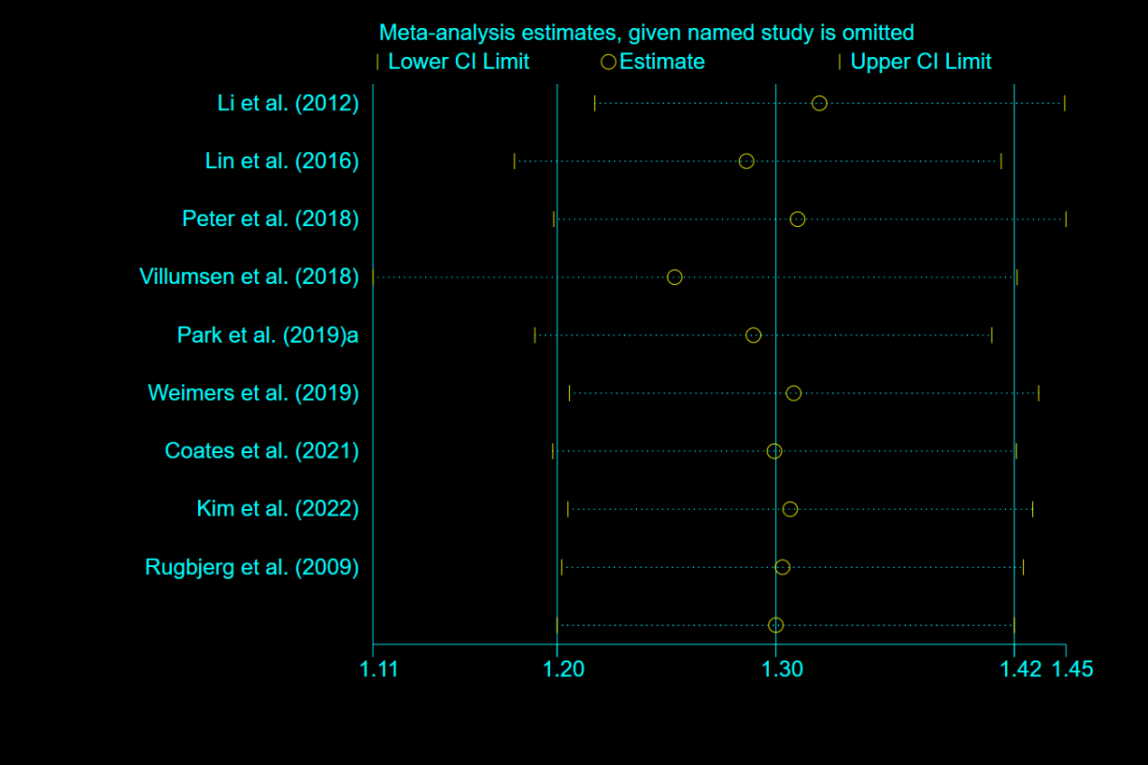
**

**D:** PD and CD

**
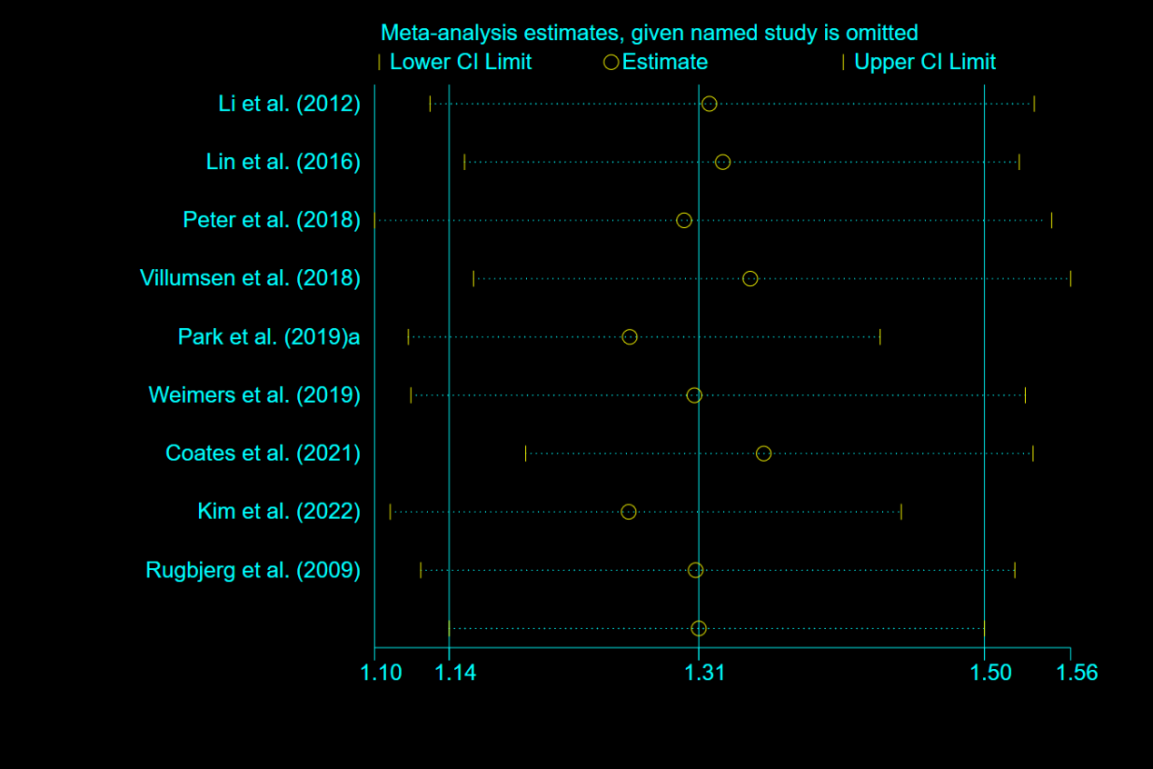
**

**E:** PD and UC

**
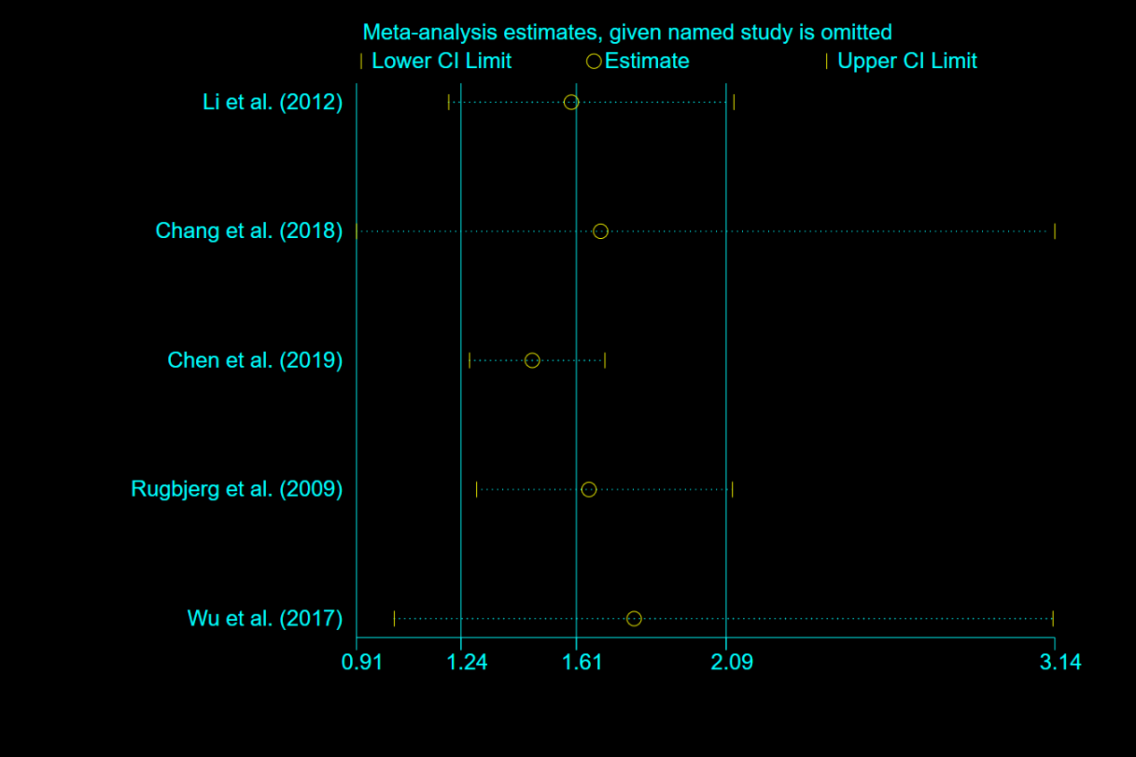
**

**F:** PD and SS

**
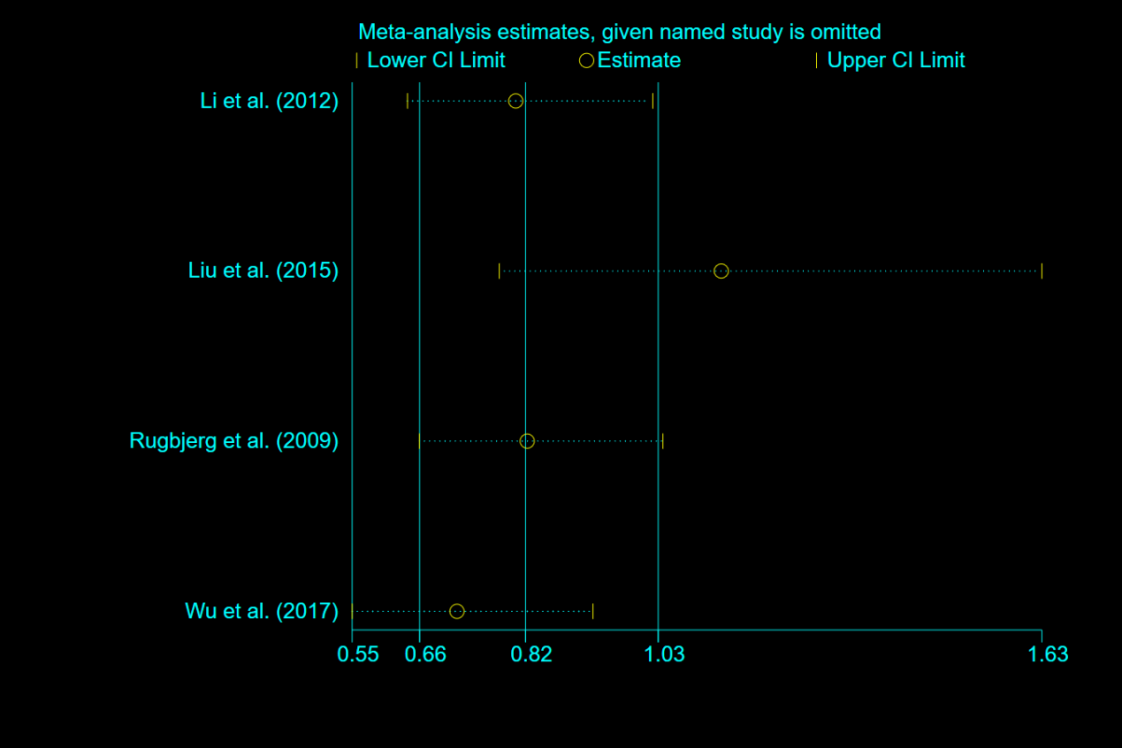
**

**G:** PD and SLE

**
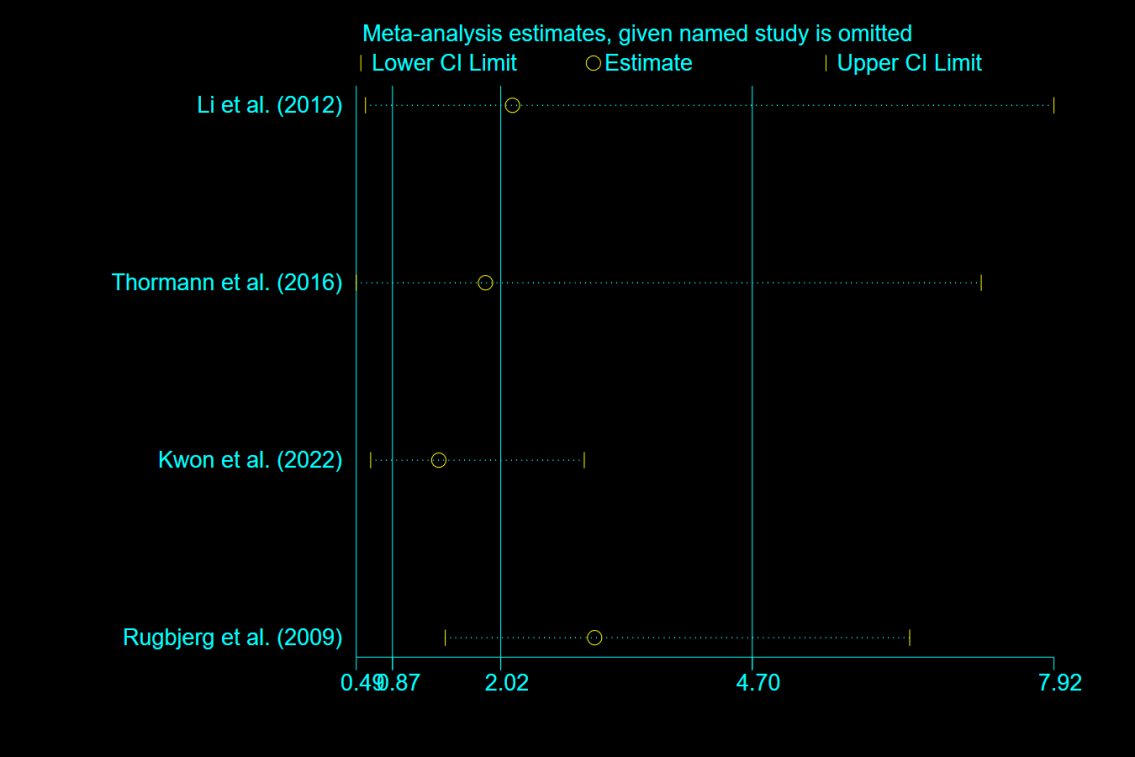
**

**H:** PD and MS

**
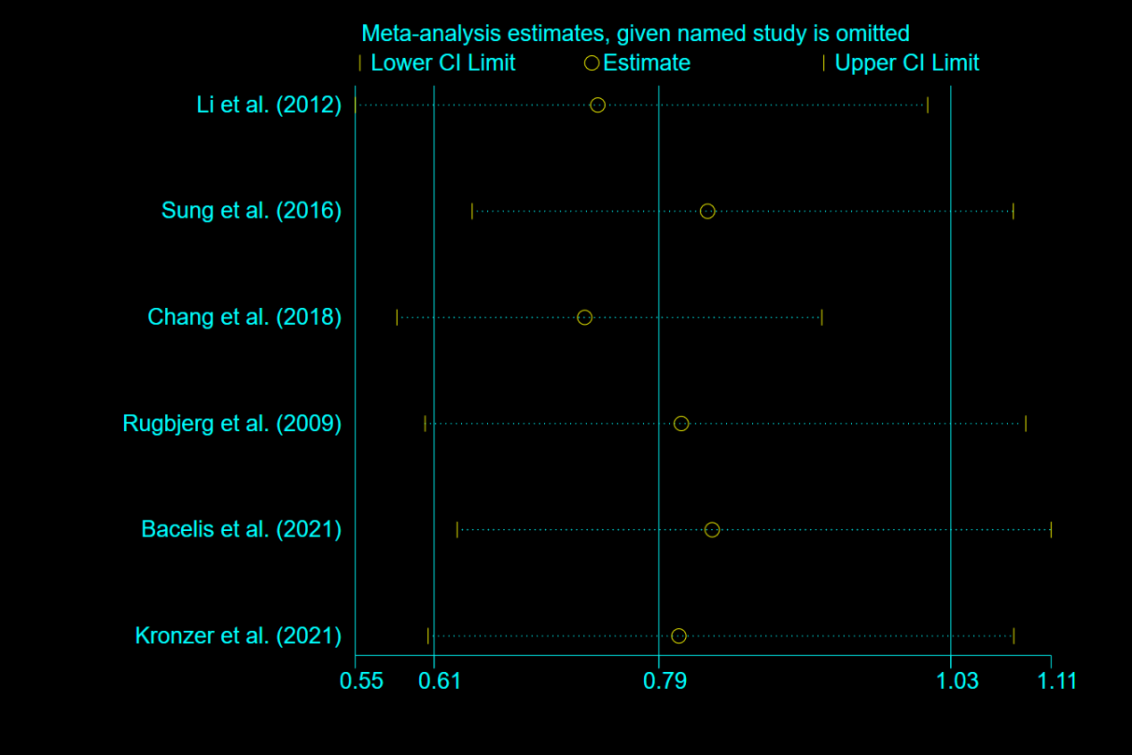
**

**I:** PD and RA

**
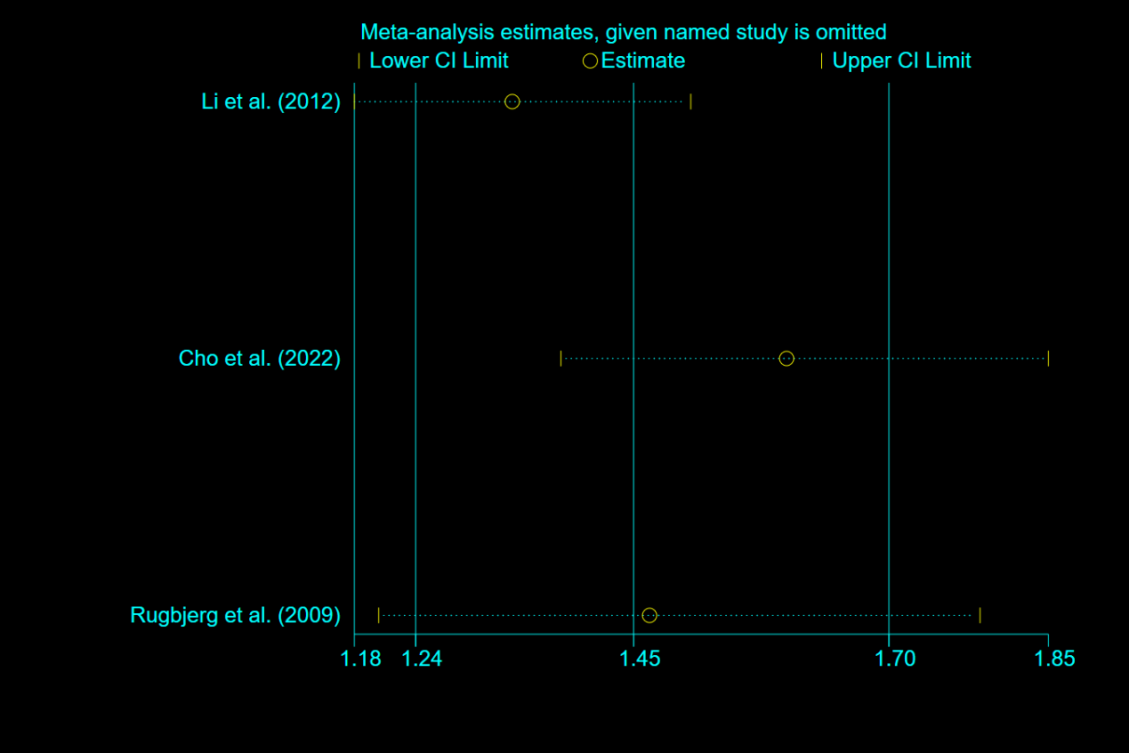
**

**J:** PD and GD

**
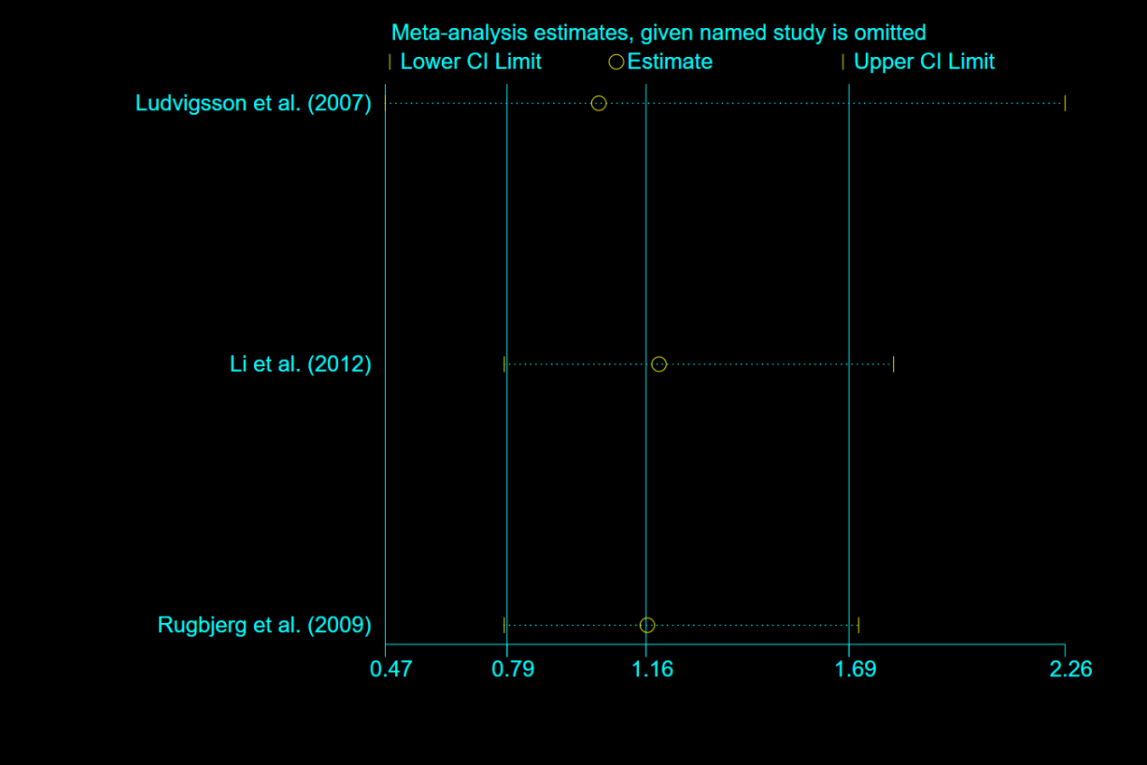
**

**K:** PD and CLD
